# Supplementary material for: Annulation of Perimidines with 5-Alkynylpyrimidines en Route to 7-Formyl-1,3-Diazopyrenes
Source: Int J Mol Sci. 2022 Dec 10;23(24):15657. doi: 10.3390/ijms232415657 (PMC9778996; doi:10.3390/ijms232415657)
Supplement: Supplementary file 1 [file ijms-23-15657-s001.zip › ijms-2024817-supplementary.pdf]

Electronic Supplementary Material (ESI)

Annulation of Perimidines with 5-Alkynylpyrimidines en route to 7-Formyl-1,3-diazopyrenes

Stanislav V. Shcherbakov[1,*], Alexander V. Aksenov[1], Maksim V. Vendin[1], Viktoria Yu. Shcherbakova[1], Anna Yu. Ivanova[1], Maksim O. Shcheglov[1], Sergei N. Ovcharov[1], and Michael Rubin[1,2,*]

1 Department of Chemistry, North Caucasus Federal University, 1a Pushkin St., 355017 Stavropol, Russia
2 Department of Chemistry, University of Kansas, 1567 Irving Hill Road, Lawrence, KS 66045, USA
* Correspondence: shcherbakov.s@gmail.com (S.V.S.); mrubin@ku.edu (M.R.)

Supporting Information

NMR Spectral Charts........ S2
^{1}H and ^{13}C NMR spectral charts for 6-benzylidene-6,10,10a,10b-tetrahydroquinazolino[6,7,8-*gh*]perimidine (14)........ S2
^{1}H and ^{13}C NMR spectral charts for 6-hexyllbenzo[*gh*]perimidine-7-carbaldehyde (16) and 6-(benzylamino)benzo[*gh*]perimidine-7-carbaldehyde (17)........ S6
HRMS spectral charts S34
HRMS spectral charts for 6-benzylidene-6,10,10a,10b-tetrahydroquinazolino[6,7,8-*gh*]perimidine (14)........ S34
HRMS spectral charts for 6-hexyllbenzo[*gh*]perimidine-7-carbaldehyde (16) and 6-(benzylamino)benzo[*gh*]perimidine-7-carbaldehyde (17)........ S35
Structure elucidation for compound 17b........ S42

^{1}H and ^{13}C NMR spectral charts for 6-benzylidene-6,10,10a,10b-tetrahydroquinazolino[6,7,8-*gh*]perimidine (14)

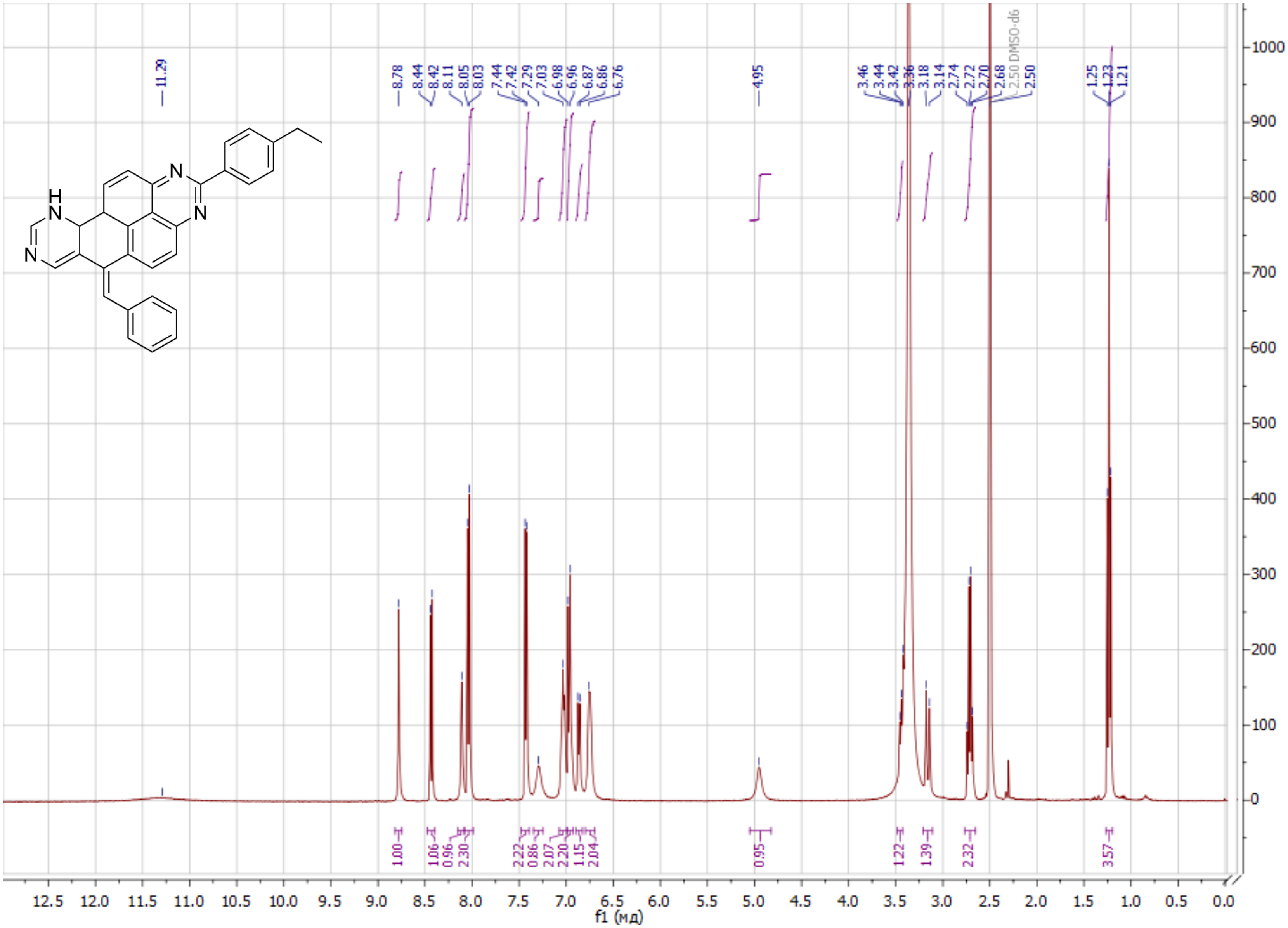

Figure S1. ^{1}H NMR spectrum of **14c** in DMSO-d_6

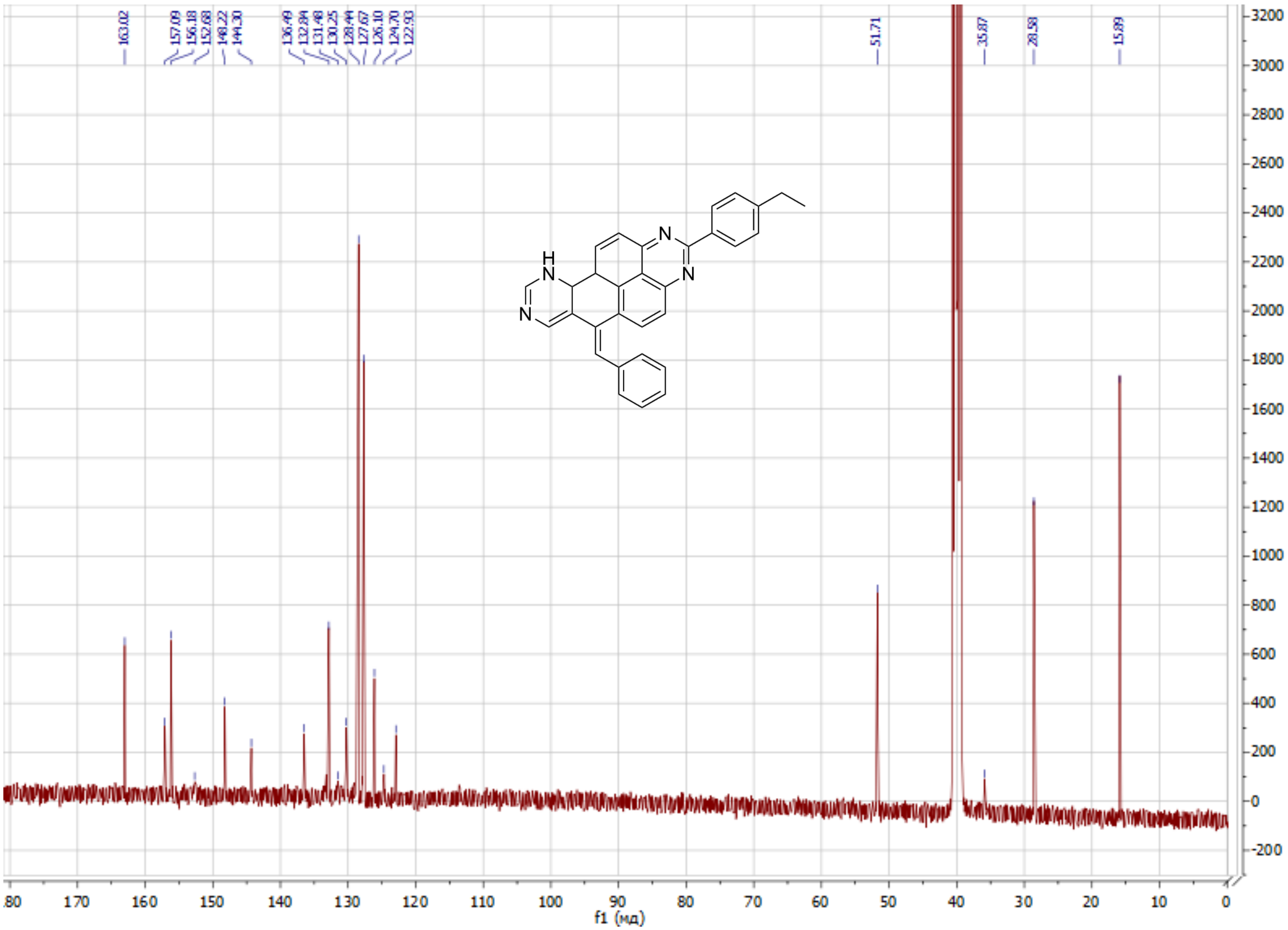

Figure S2. ^{13}C DEPTQ-135 spectrum of **14c** in DMSO-d_6

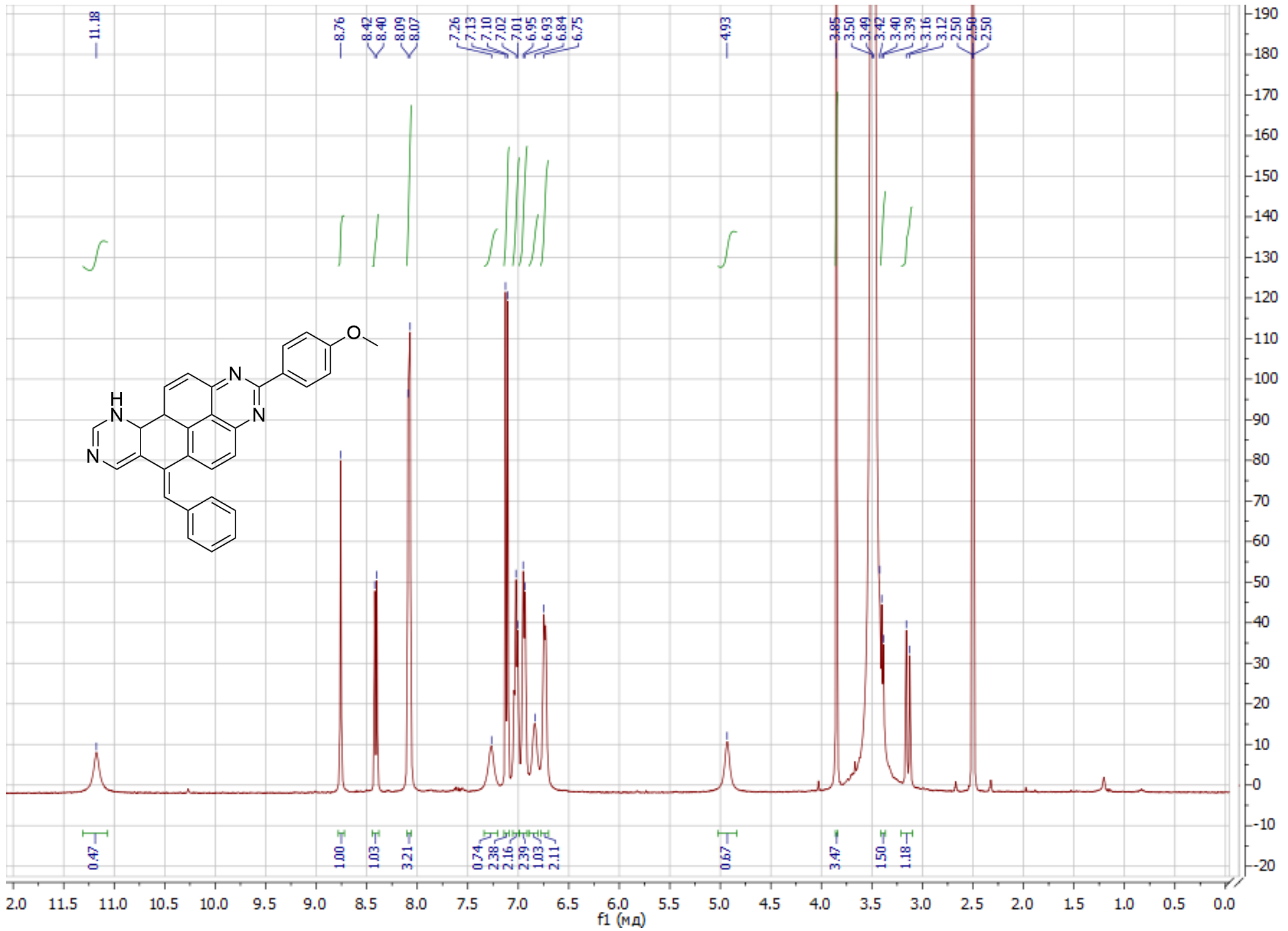

Figure S3. ^{1}H NMR spectrum of **14e** in DMSO-d_6 (101 MHz)

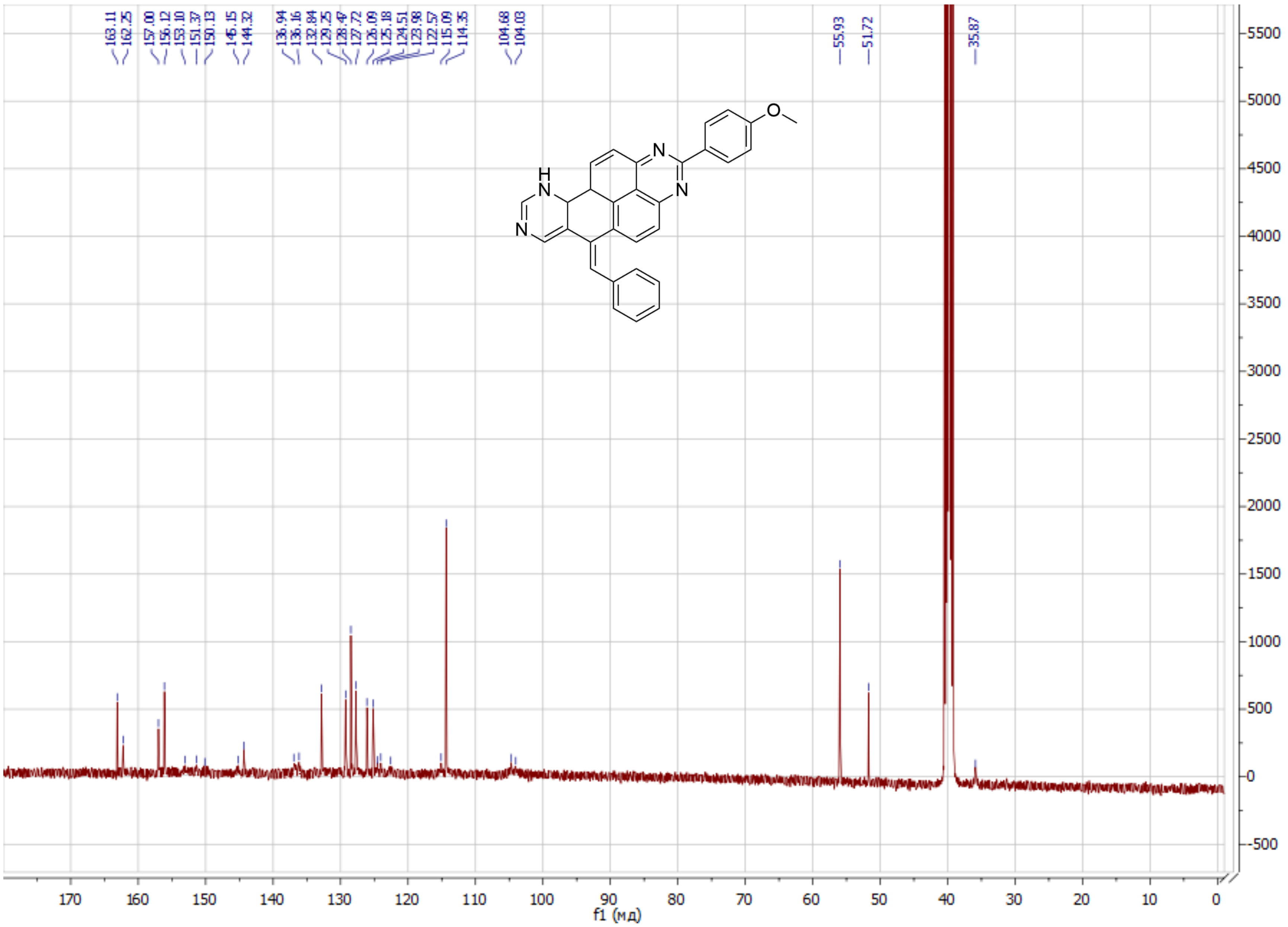

Figure S4. ^{13}C DEPTQ-135 spectrum of **14e** in DMSO-d_6 (101 MHz)

^{1}H and ^{13}C NMR spectral charts 6-hexyllbenzo[*gh*]perimidine-7-carbaldehyde (16) and 6-(benzylamino)benzo[*gh*]perimidine-7-carbaldehyde (17)

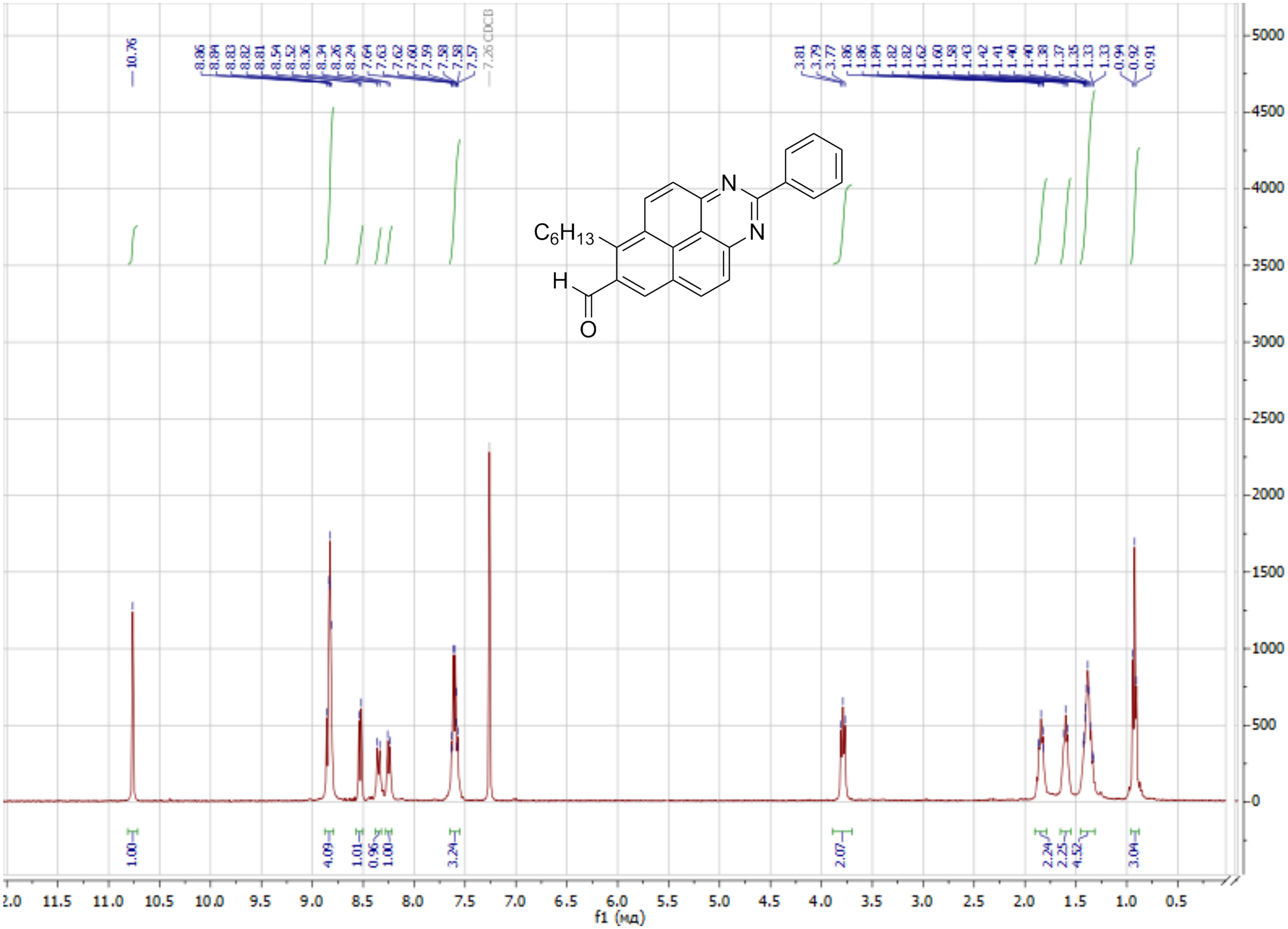

Figure S5. ^{1}H NMR spectrum of **16a** in $CDCl_3$ (400 MHz)

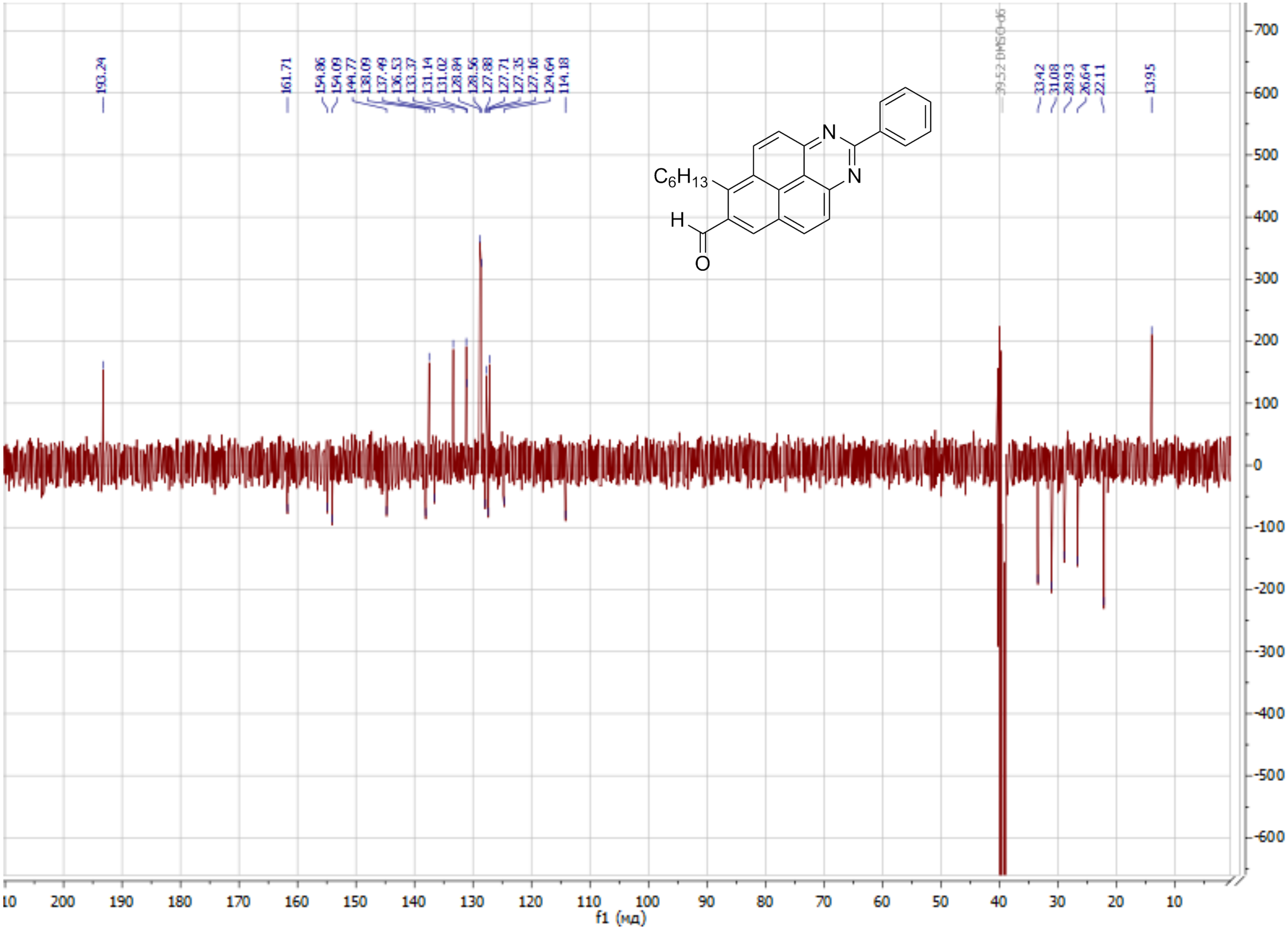

Figure S6. ^{13}C DEPTQ-135 NMR spectrum of **16a** in DMSO-d_6 (101 MHz)

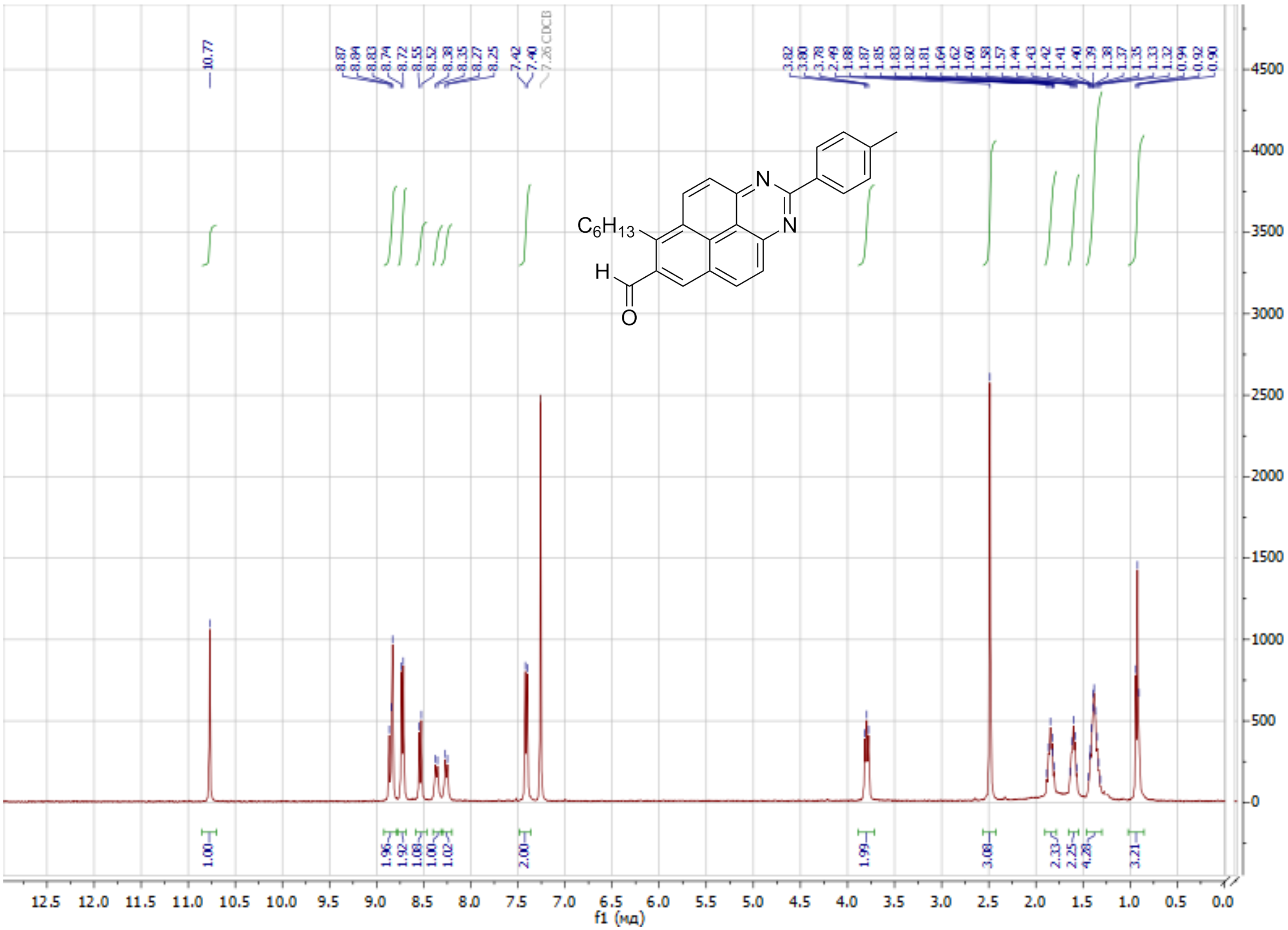

Figure S7. ^{1}H NMR spectrum of **16b** in $CDCl_3$ (400 MHz)

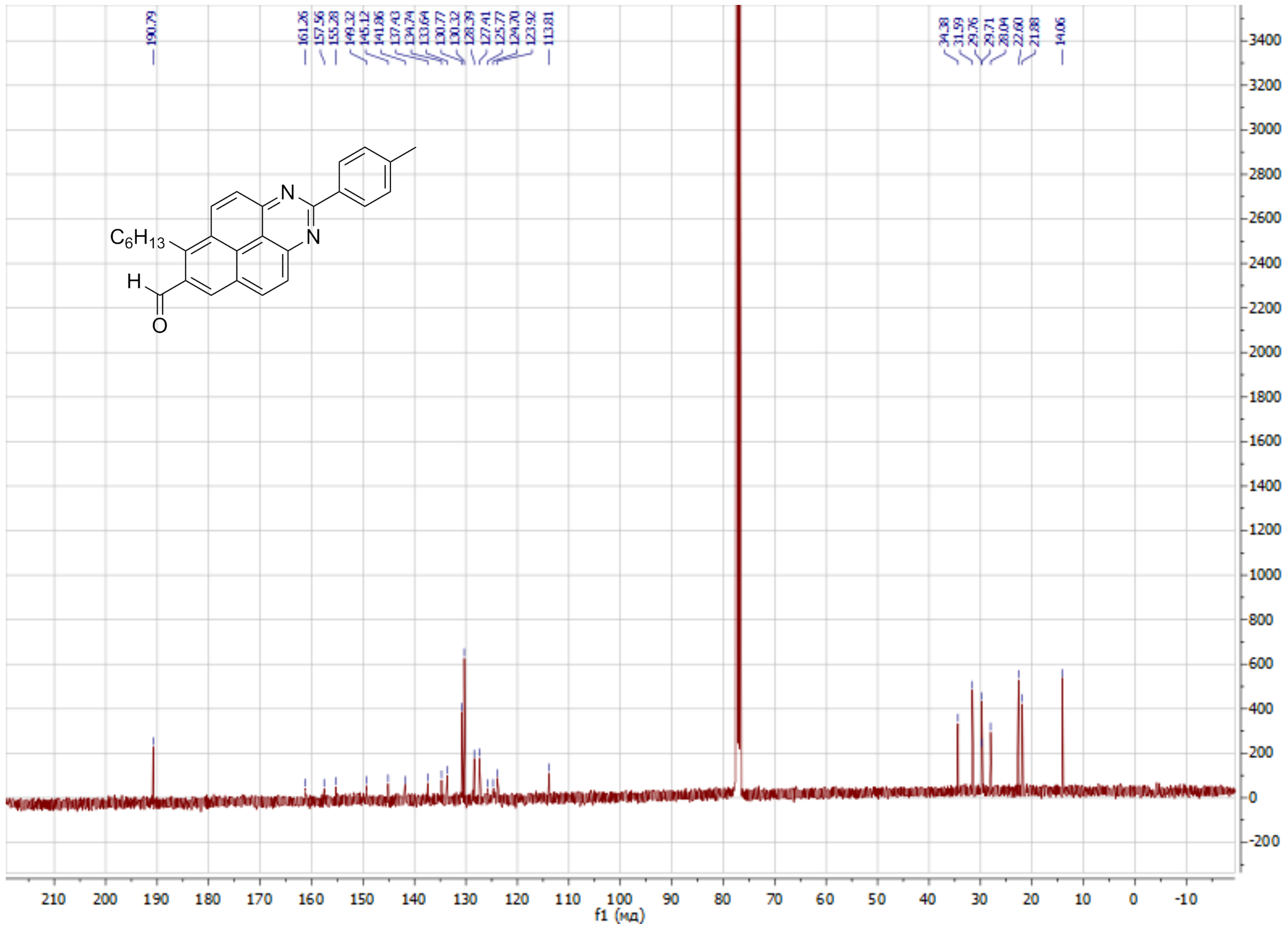

Figure S8. ^{13}C CPD NMR spectrum of **16b** in $CDCl_3$ (101 MHz)

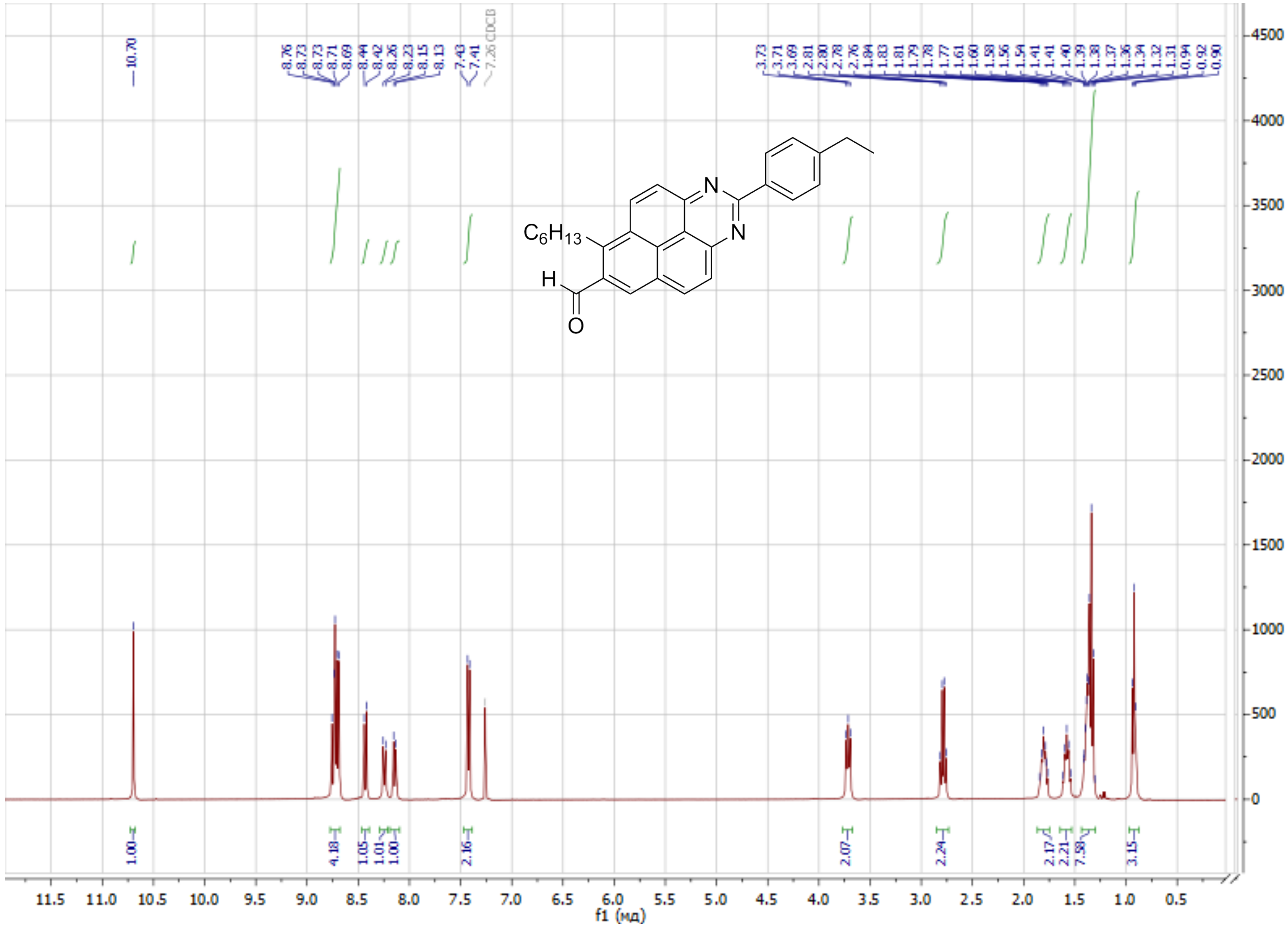

Figure S9. ^{1}H NMR spectrum of **16c** in $CDCl_3$ (400 MHz)

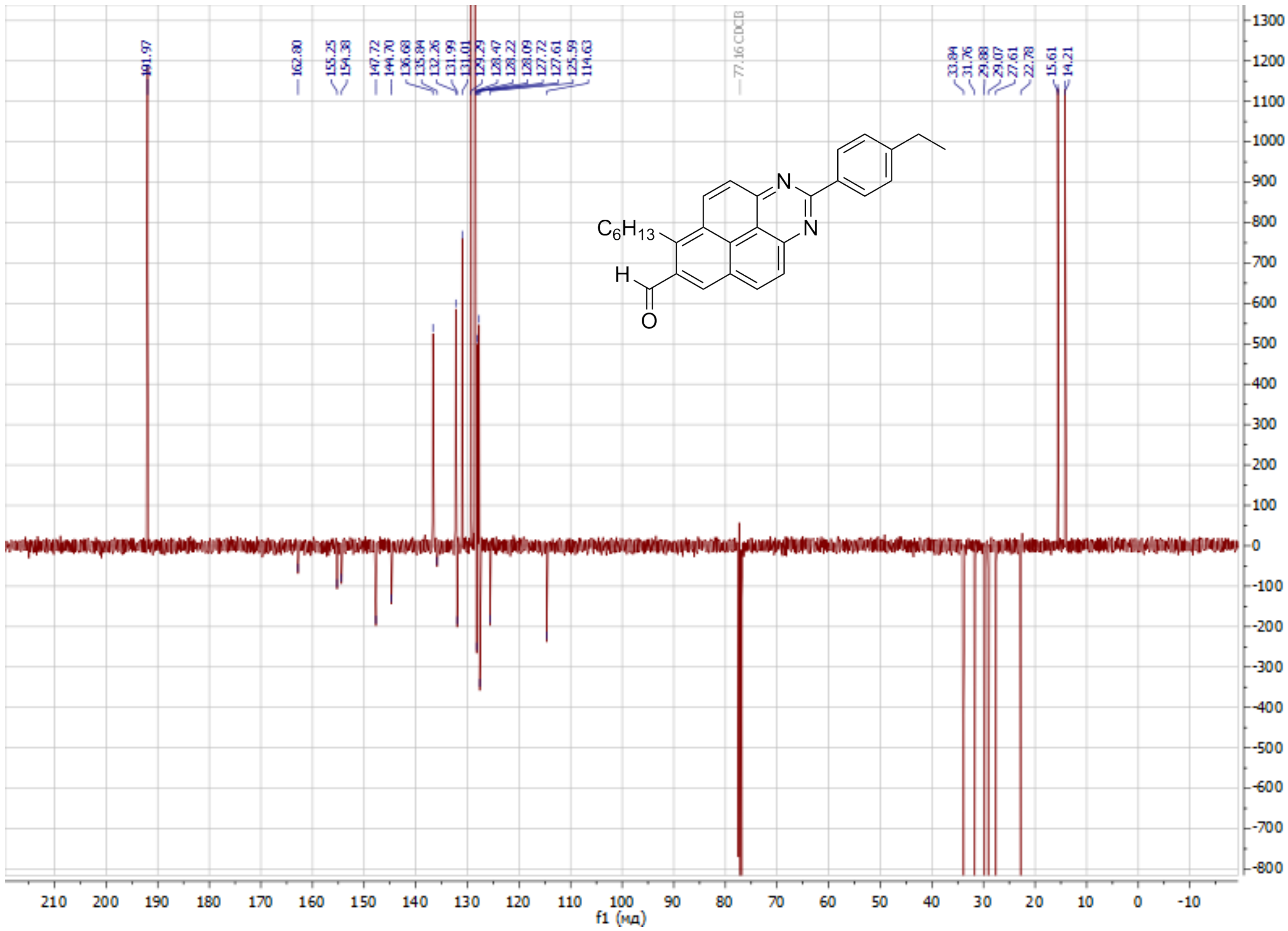

Figure S10. ^{13}C DEPTQ-135 NMR spectrum of **16c** in $CDCl_3$ (101 MHz)

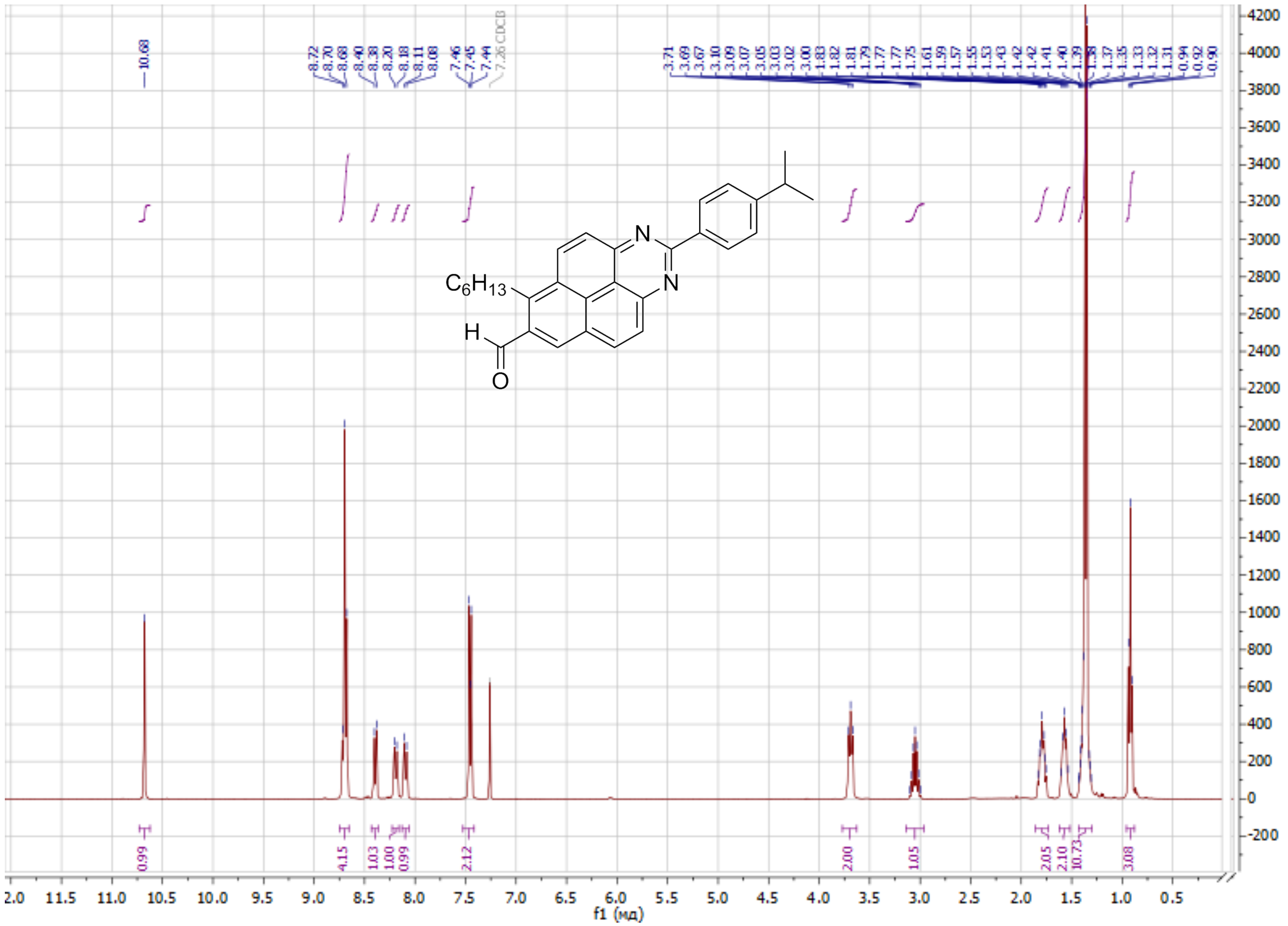

Figure S11. ^{1}H NMR spectrum of **16d** in $CDCl_3$ (400 MHz)

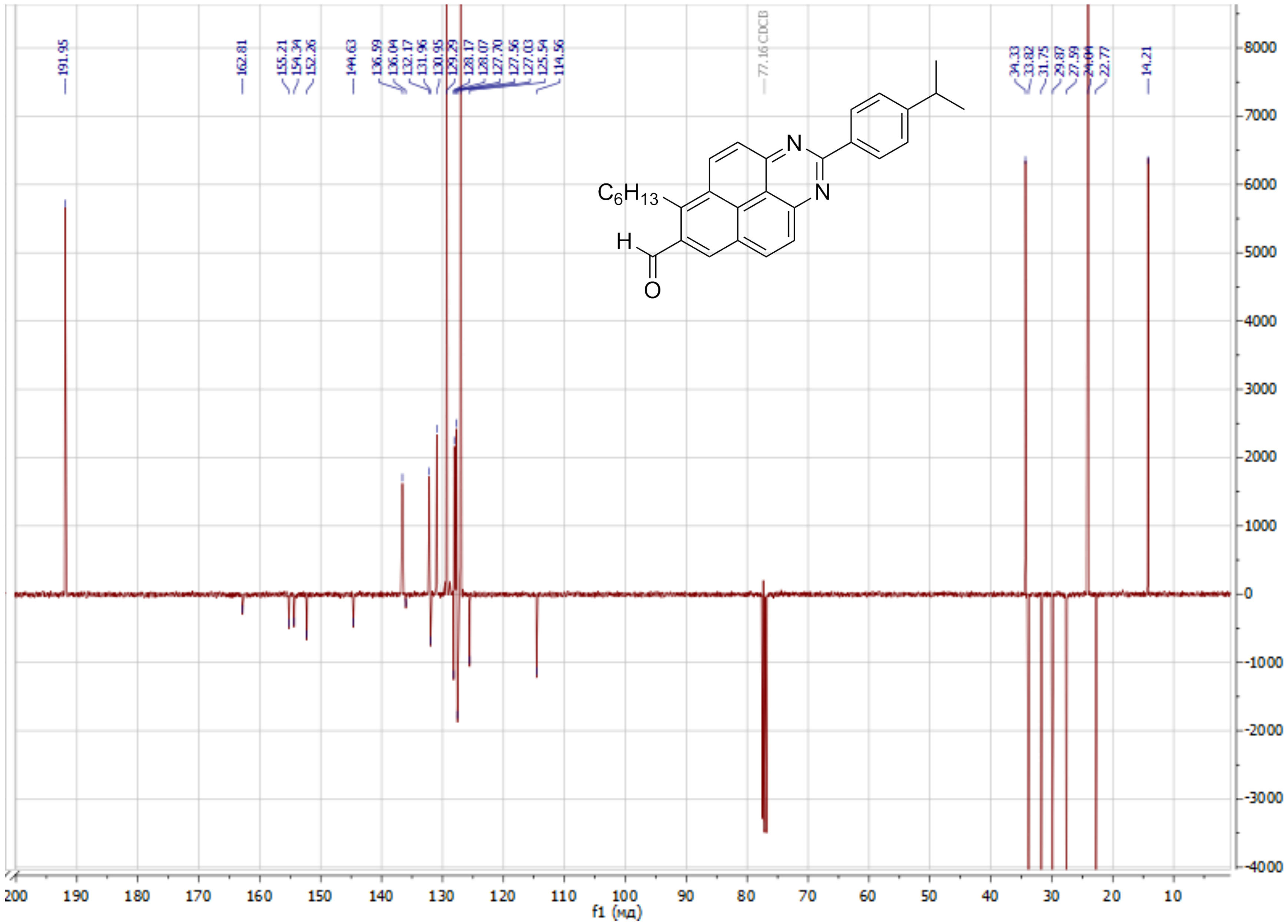

Figure S12. ^{13}C DEPTQ-135 NMR spectrum of **16d** in $CDCl_3$ (101 MHz)

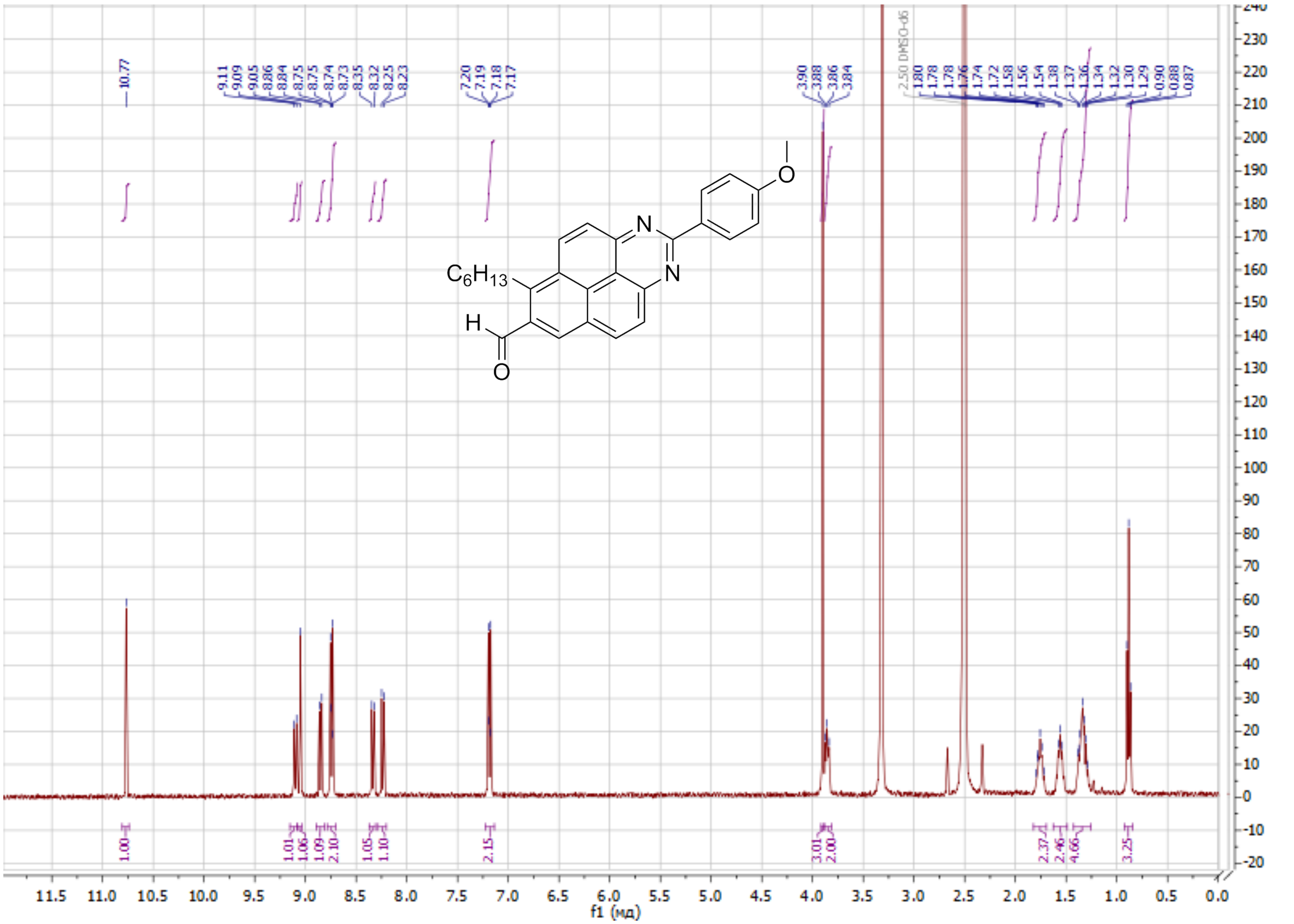

Figure S13. ^{1}H NMR spectrum of **16e** in DMSO-d_6 (400 MHz)

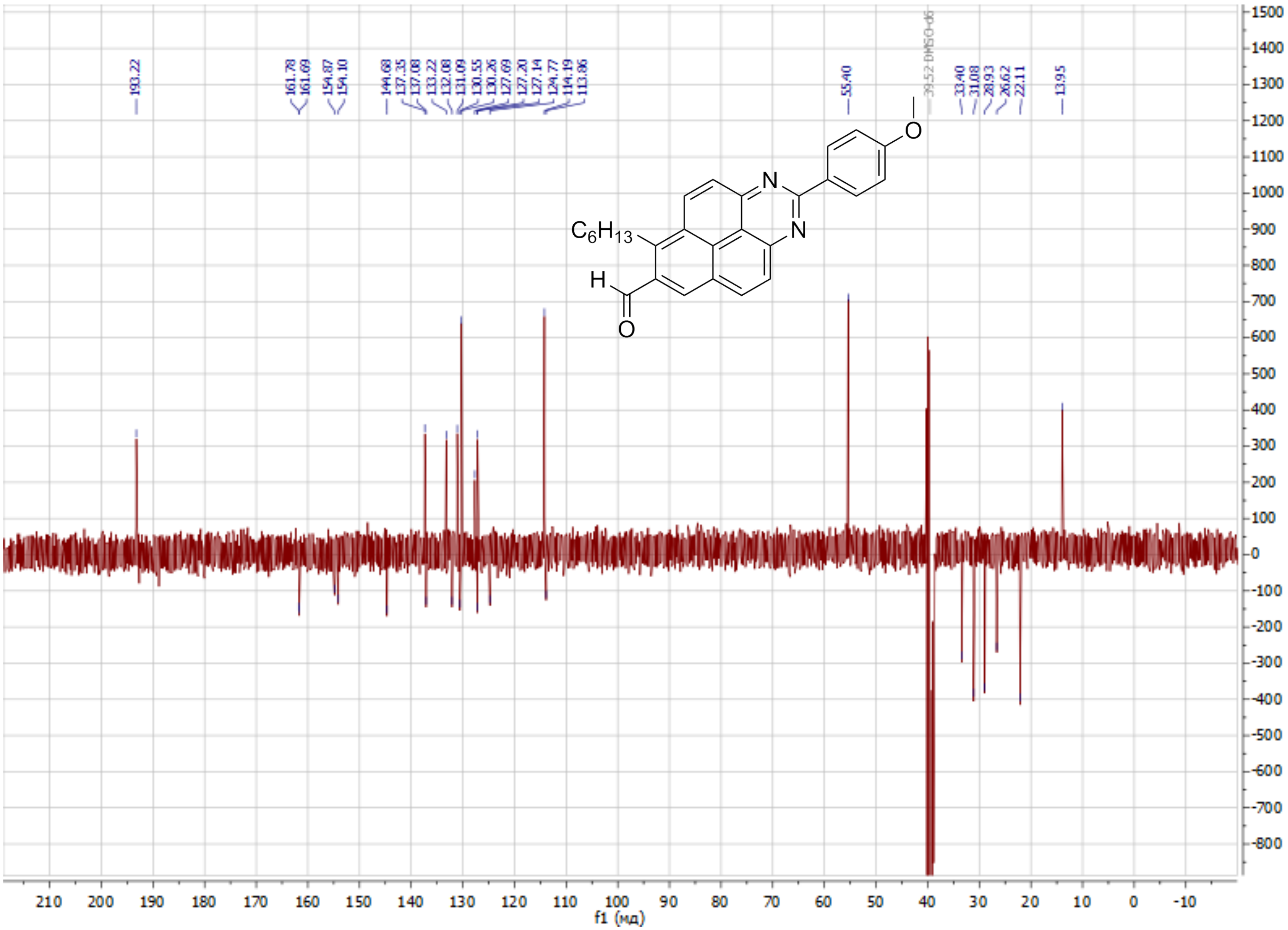

Figure S14. ^{13}C DEPTQ-135 NMR spectrum of **16e** in DMSO-d_6 (101 MHz)

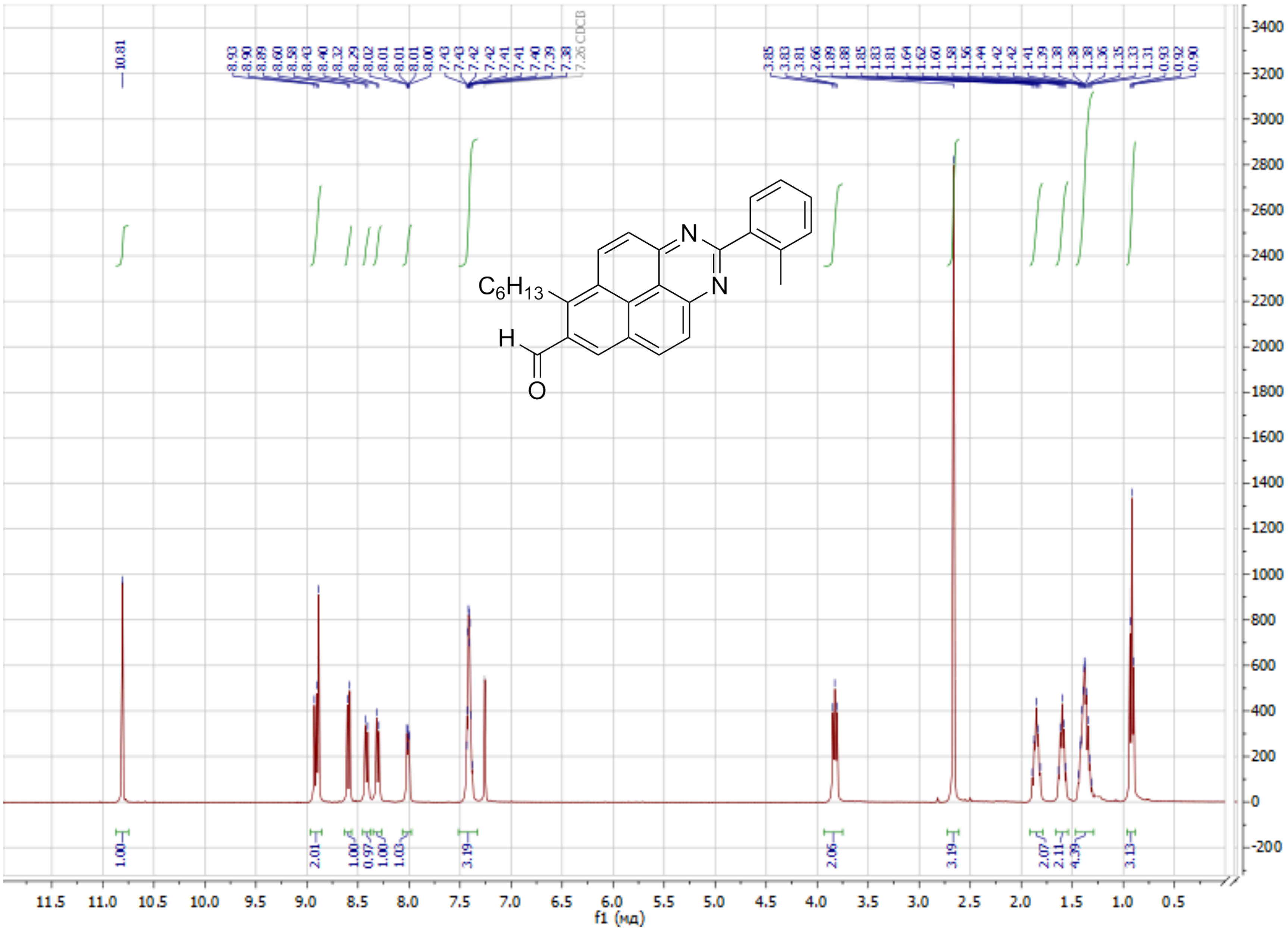

Figure S15. ^{1}H NMR spectrum of **16f** in $CDCl_3$ (400 MHz)

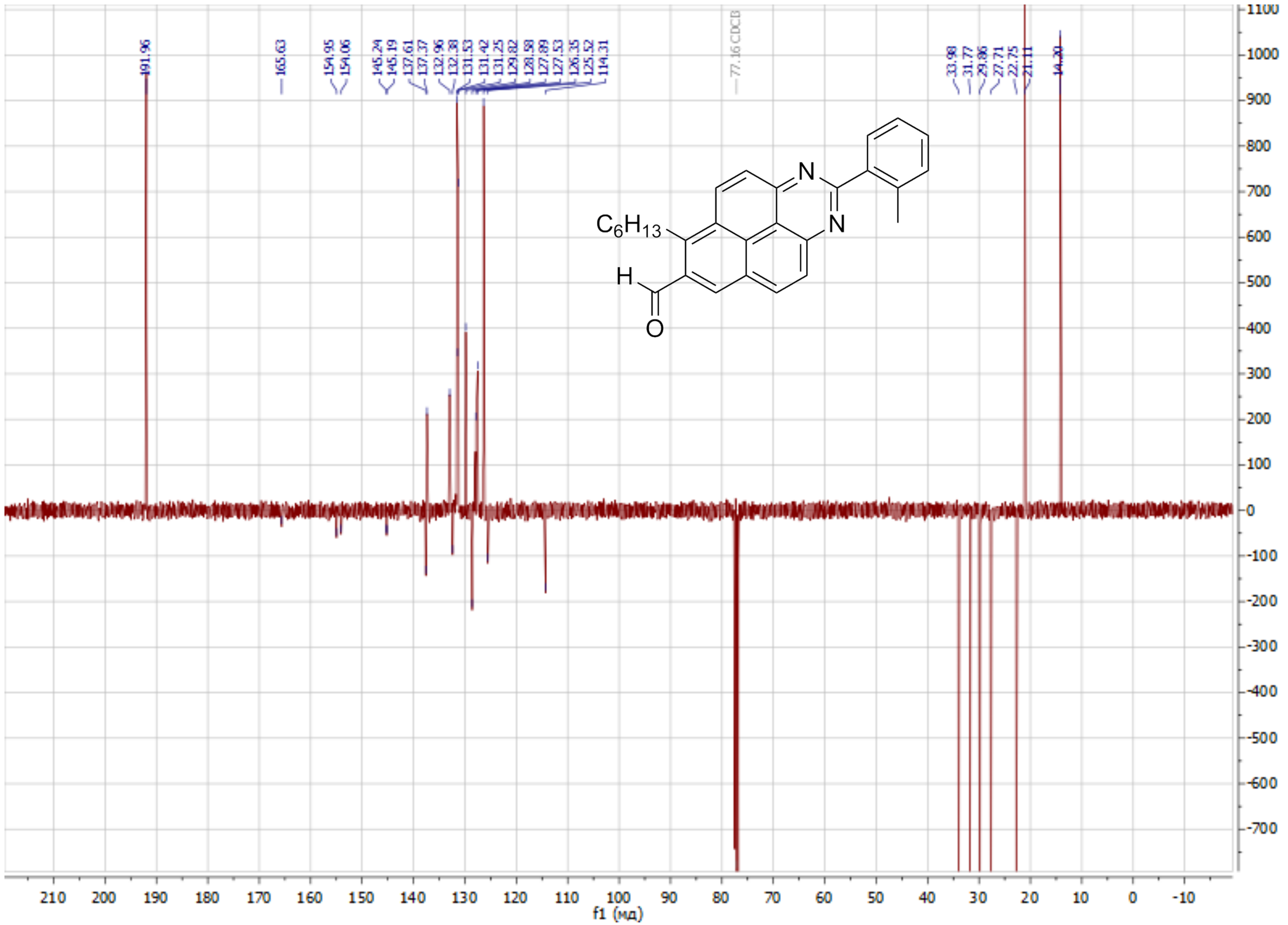

Figure S16. ^{13}C DEPTQ-135 NMR spectrum of **16f** in $CDCl_3$ (101 MHz)

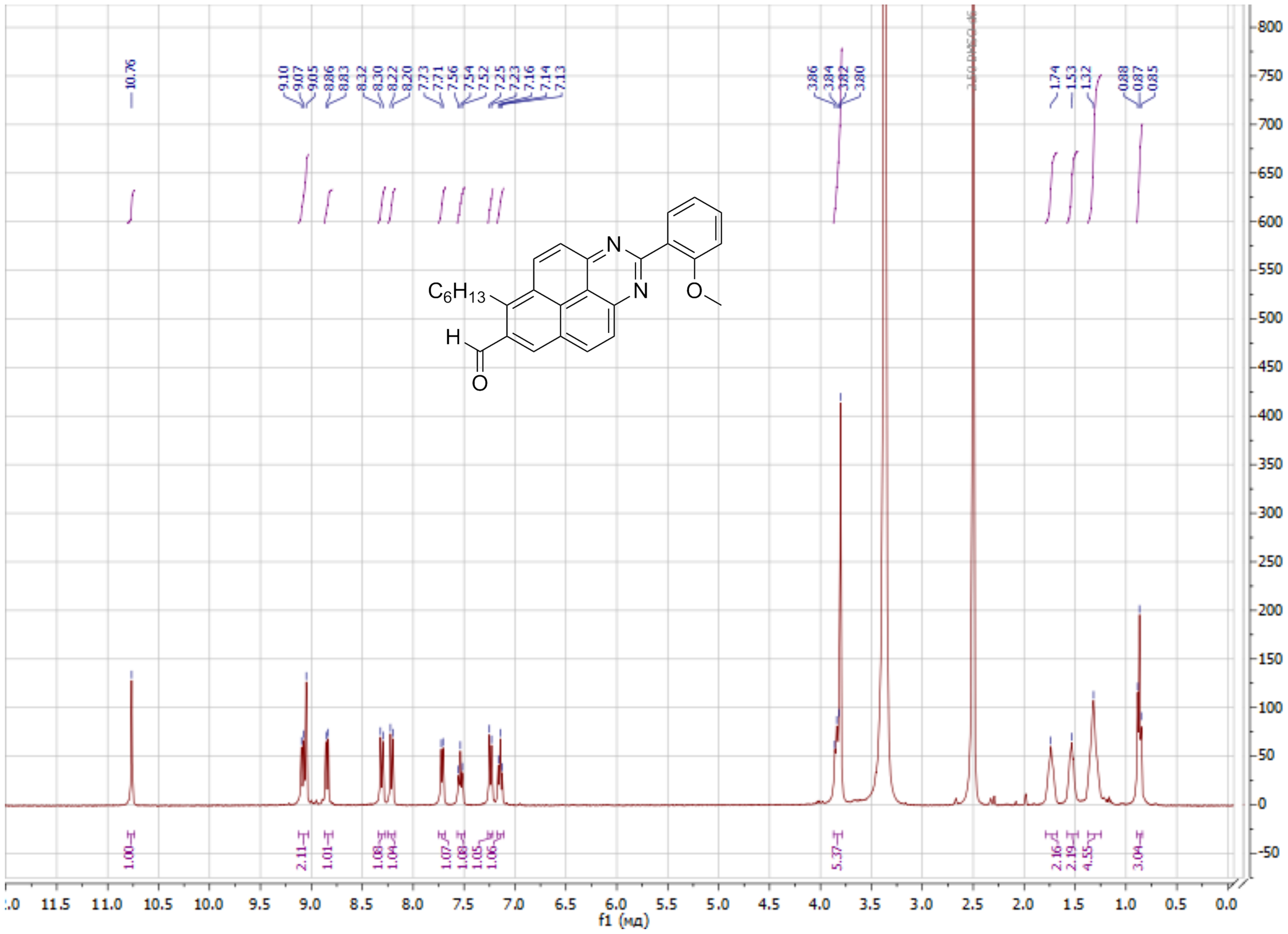

Figure S17. ^{1}H NMR spectrum of **16g** in DMSO-d_6 (400 MHz)

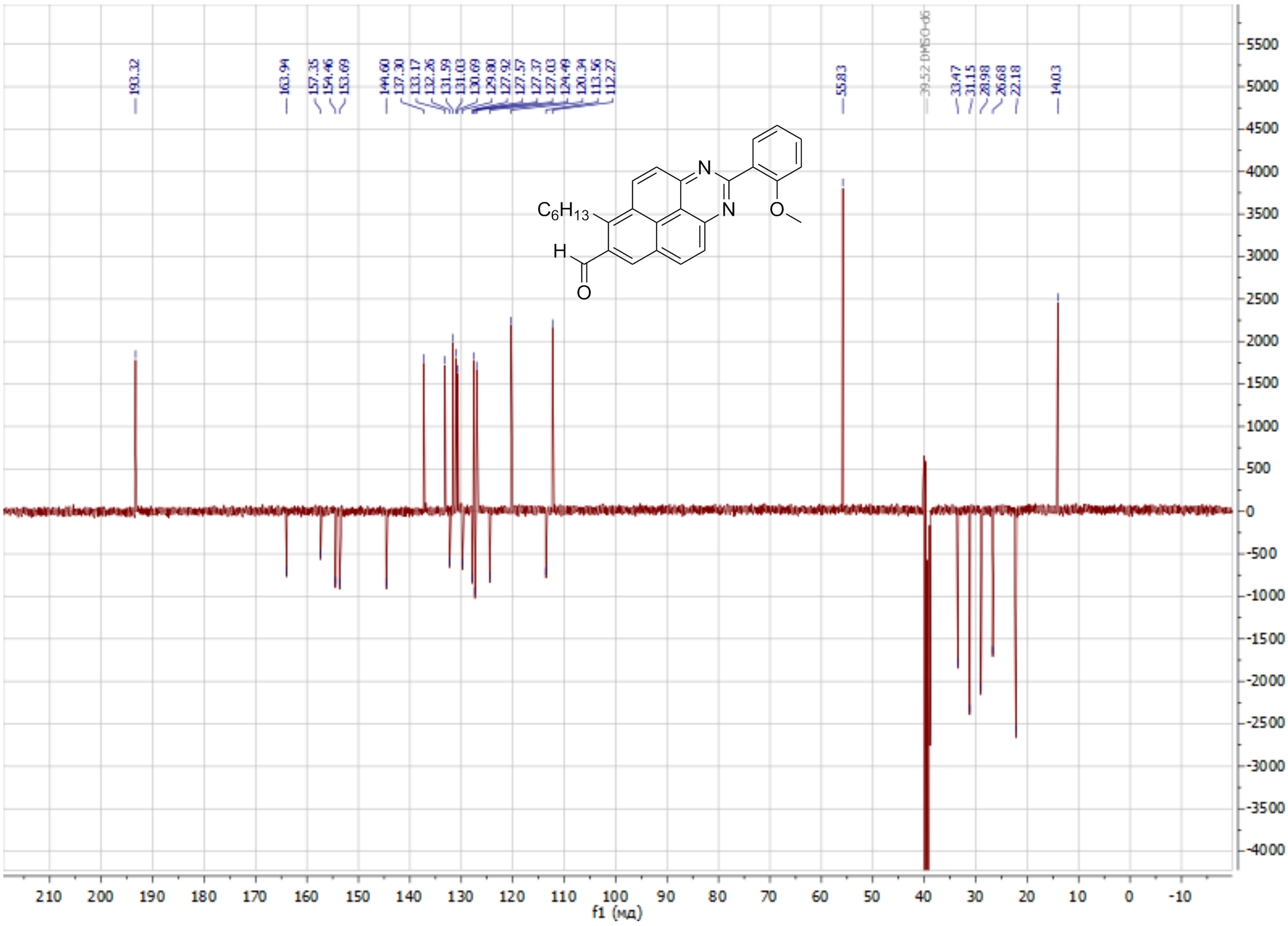

Figure S18. ^{13}C DEPTQ-135 NMR spectrum of **16g** in DMSO-d_6 (101 MHz)

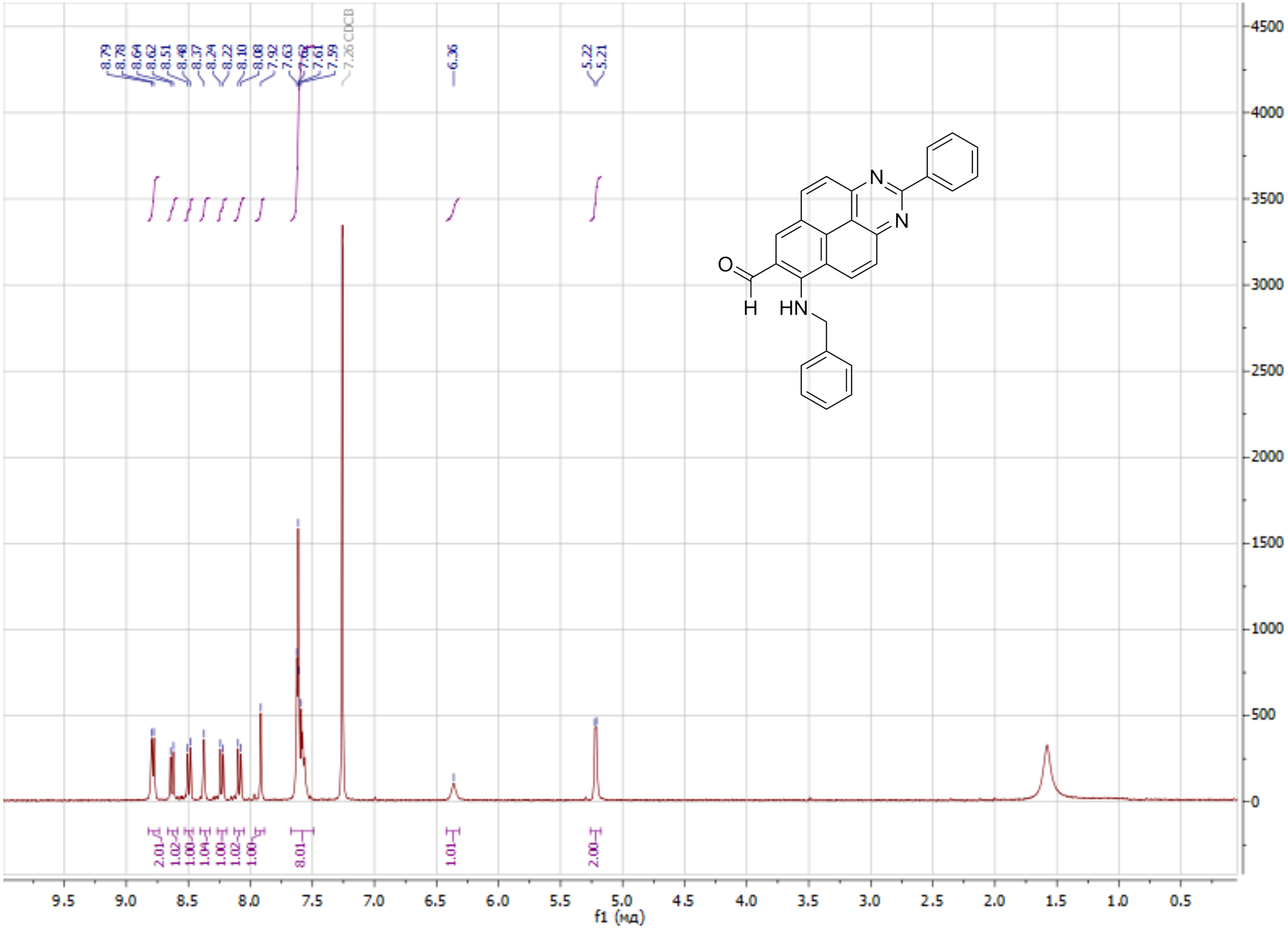

Figure S19. ^{1}H NMR spectrum of **17a** in $CDCl_3$ (400 MHz)

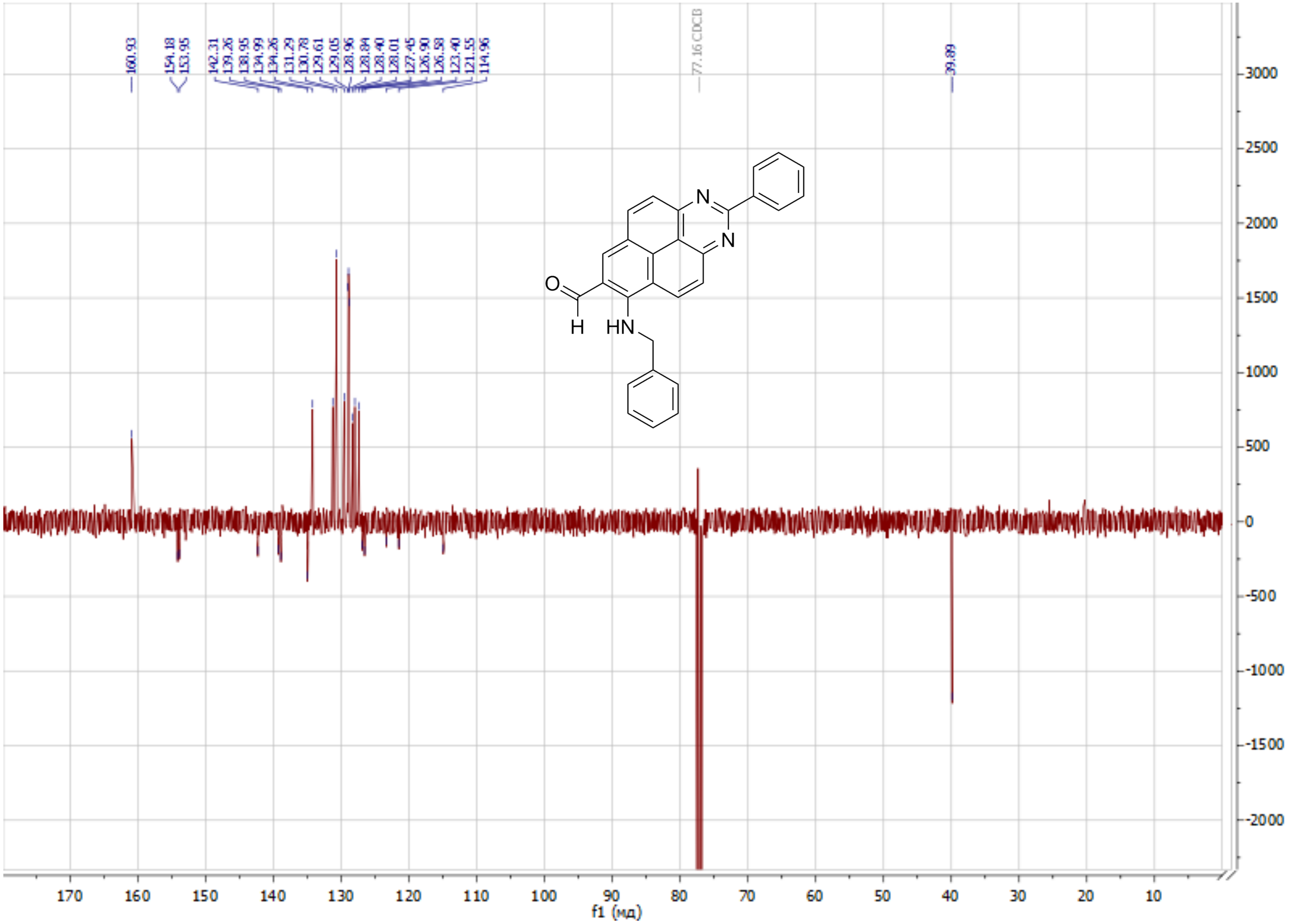

Figure S20. ^{13}C DEPTQ-135 NMR spectrum of **17a** in $CDCl_3$ (101 MHz)

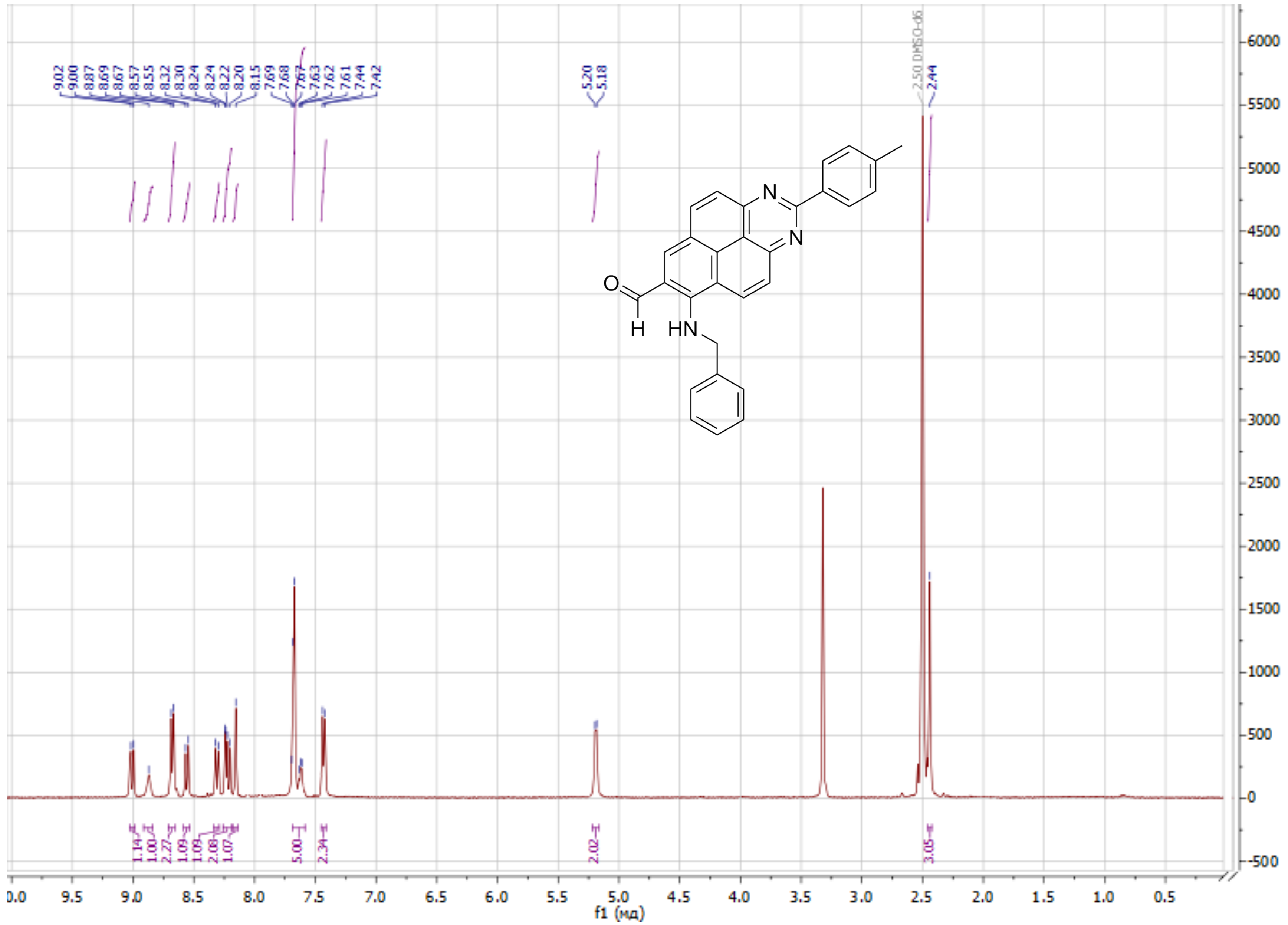

Figure S21. ^{1}H NMR spectrum of **17b** in DMSO-d_6 (400 MHz)

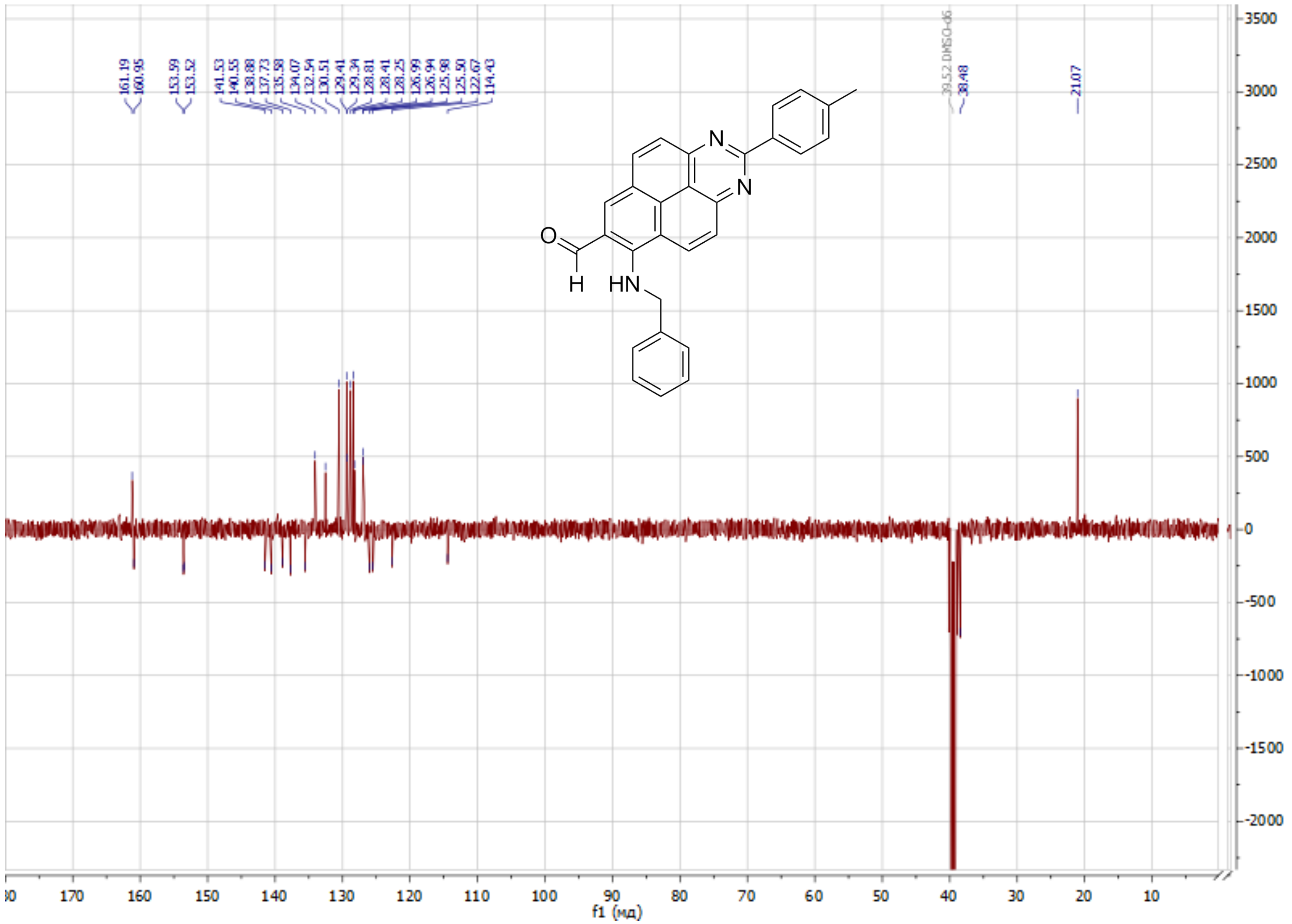

Figure S22. ^{13}C DEPTQ-135 NMR spectrum of **17b** in DMSO-d_6 (101 MHz)

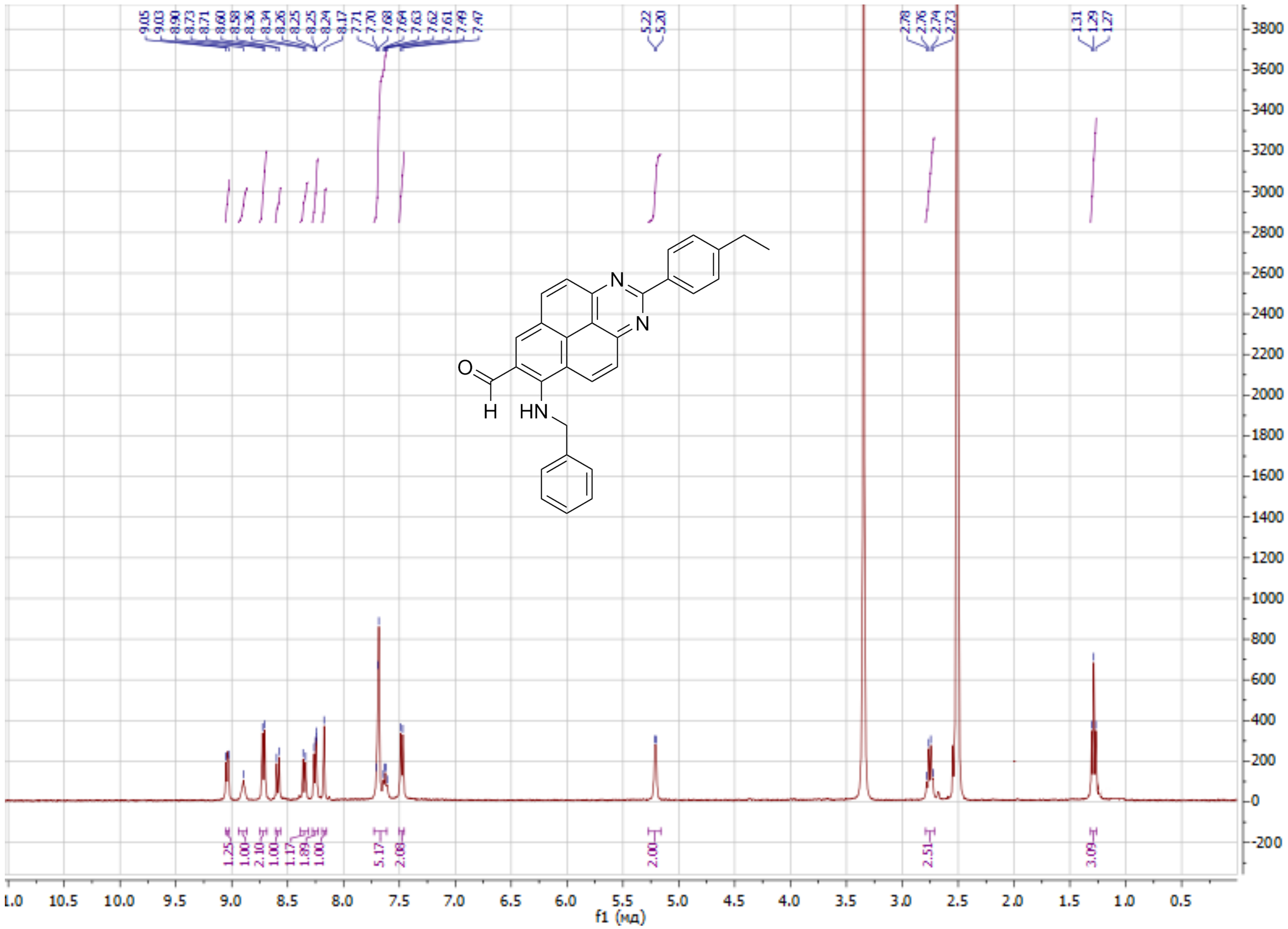

Figure S23. ^{1}H NMR spectrum of **17c** in DMSO-d_6 (400 MHz)

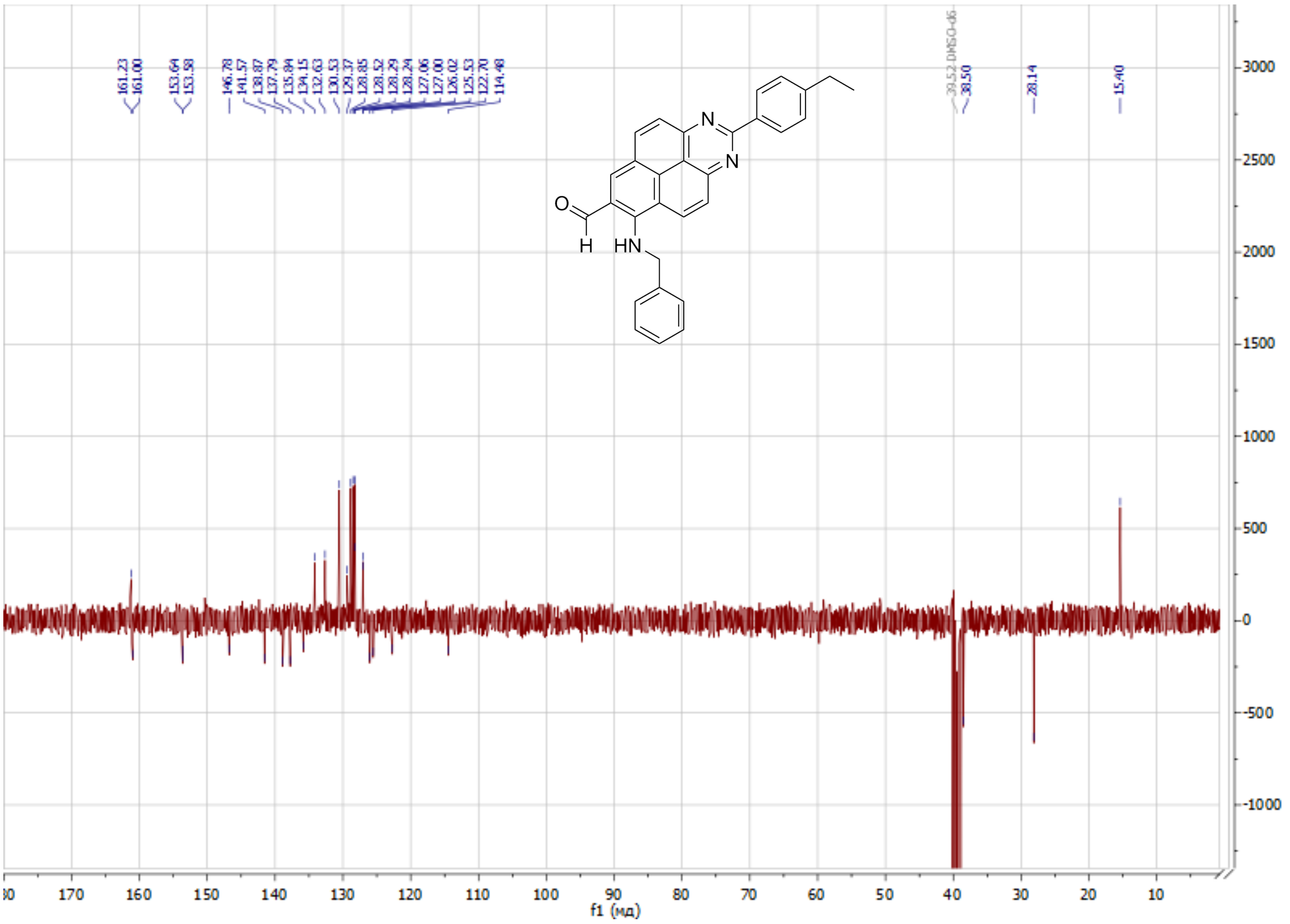

Figure S24. ^{13}C DEPTQ-135 NMR spectrum of **17c** in DMSO-d_6 (101 MHz)

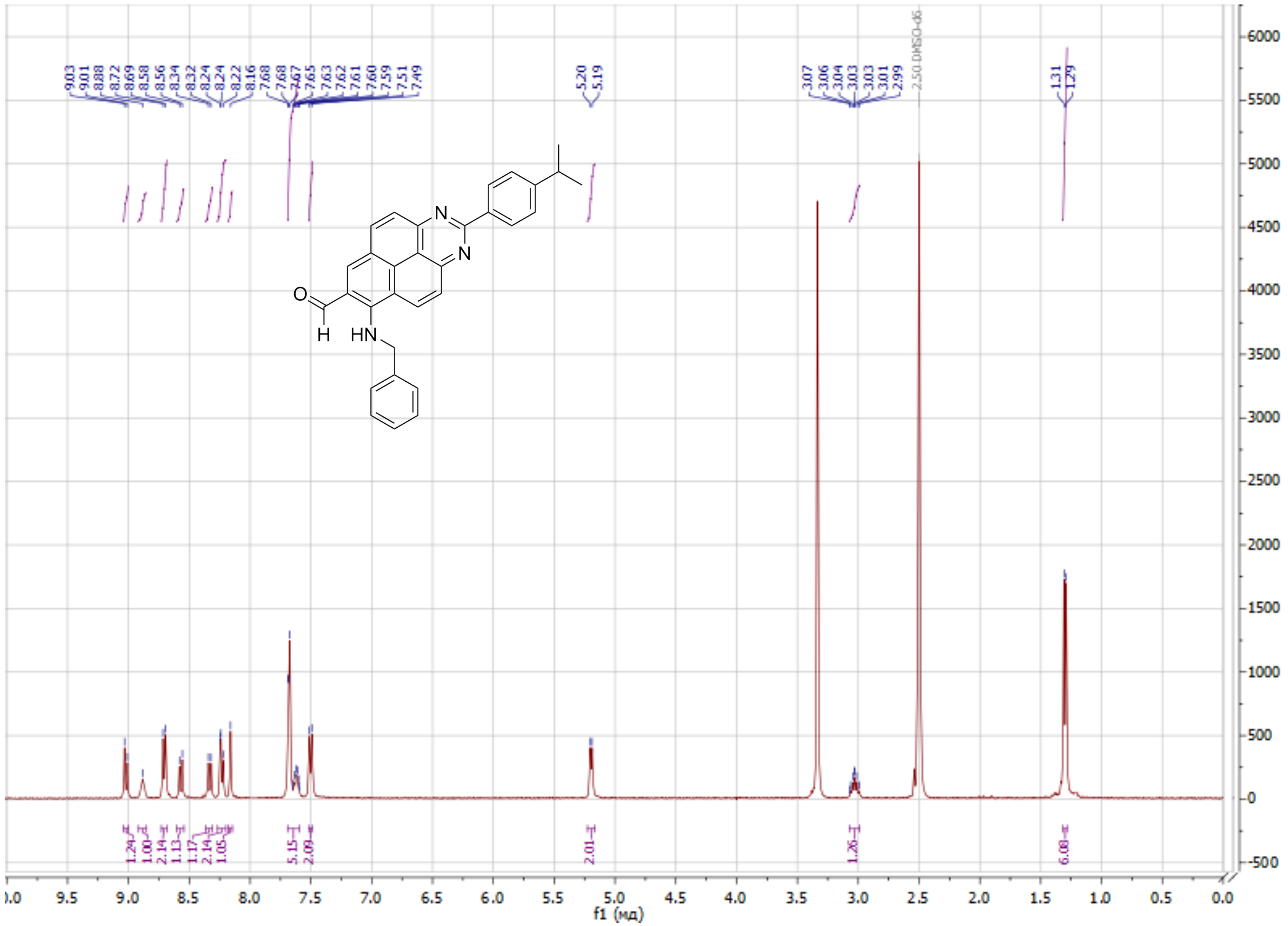

Figure S25. ^{1}H NMR spectrum of **17d** in DMSO-d_6 (400 MHz)

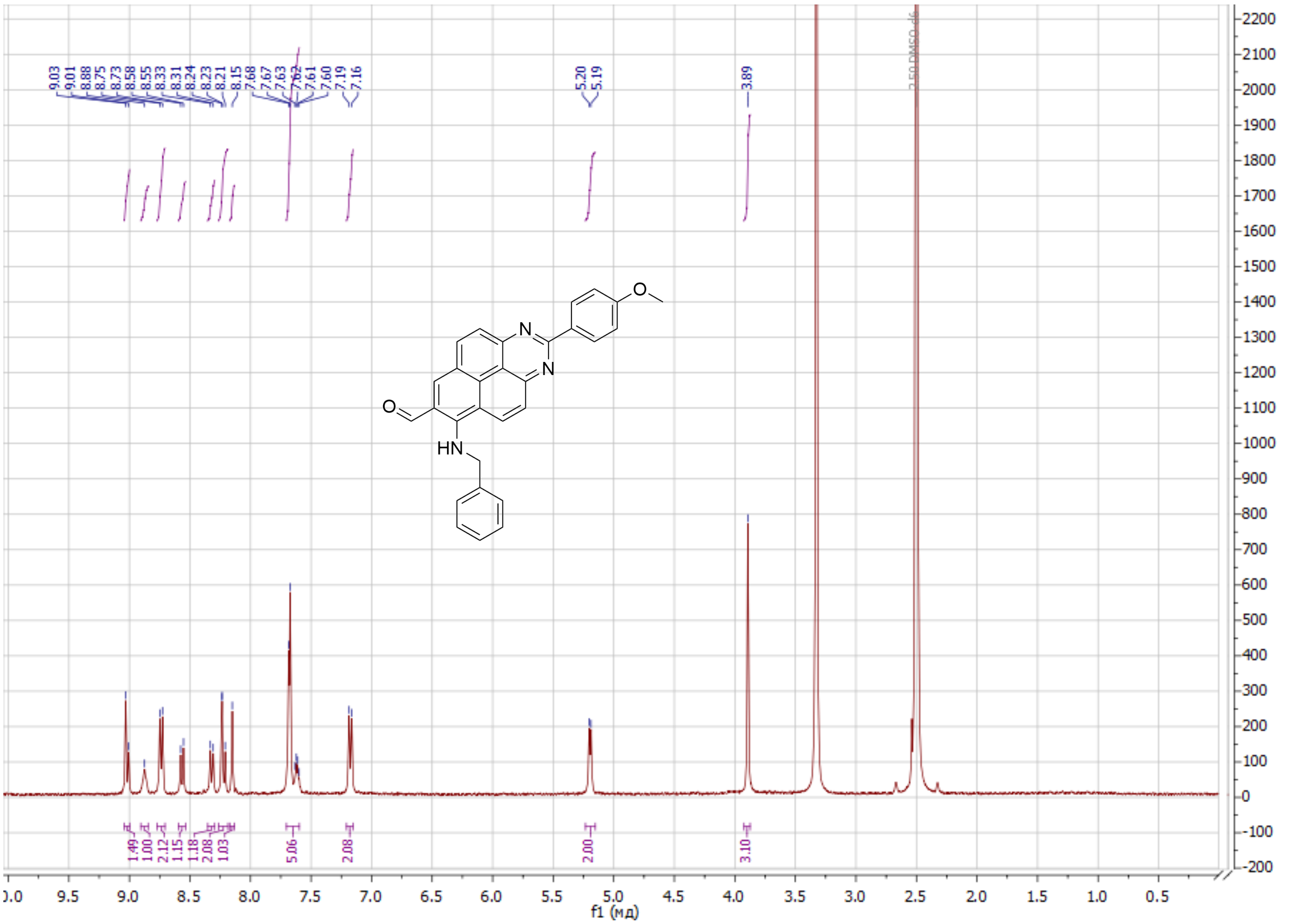

Figure S26. ^{1}H NMR spectrum of **17e** in DMSO-d_6 (101 MHz)

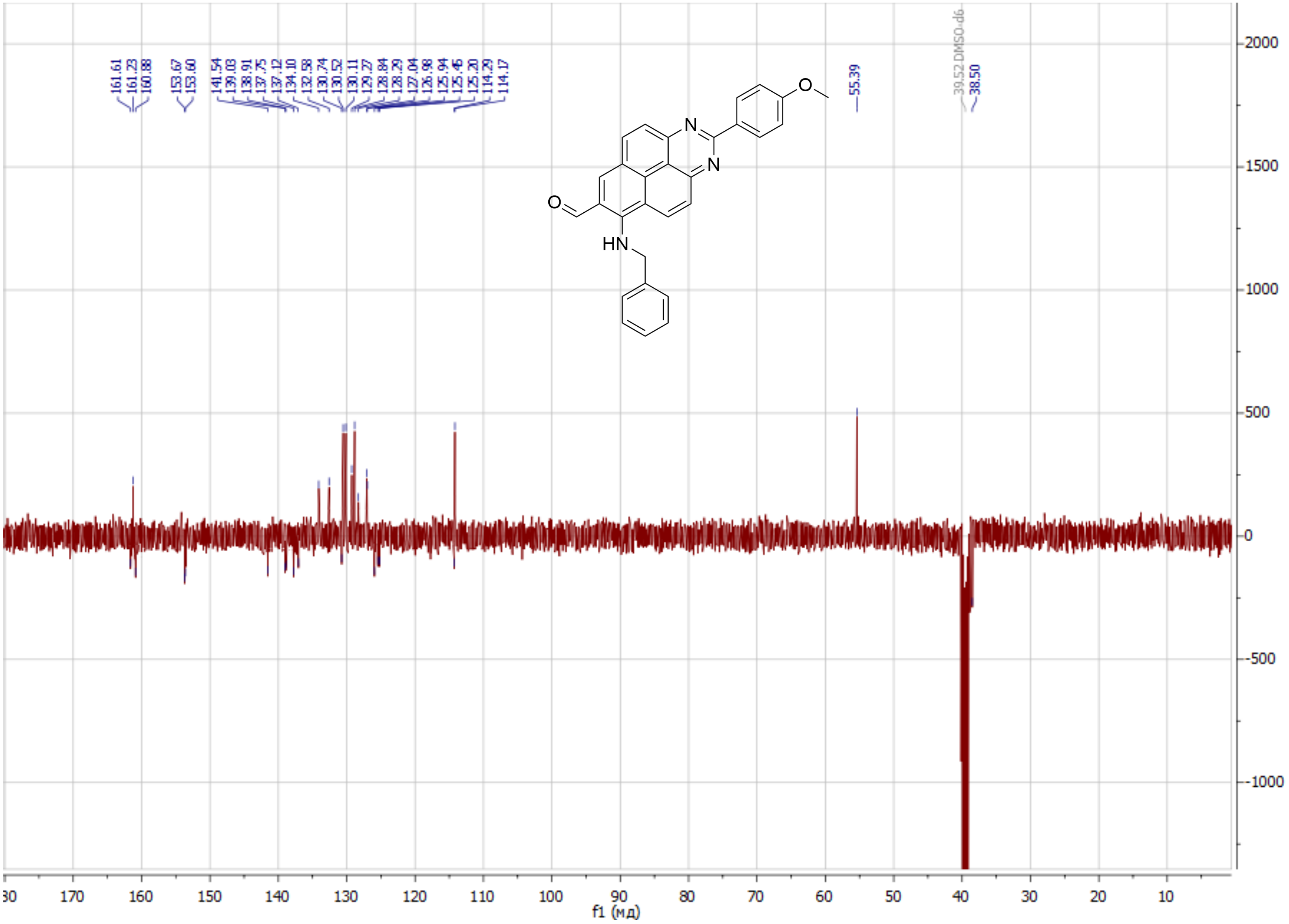

Figure S27. ^{13}C DEPTQ-135 NMR spectrum of **17e** in DMSO-d_6 (101 MHz)

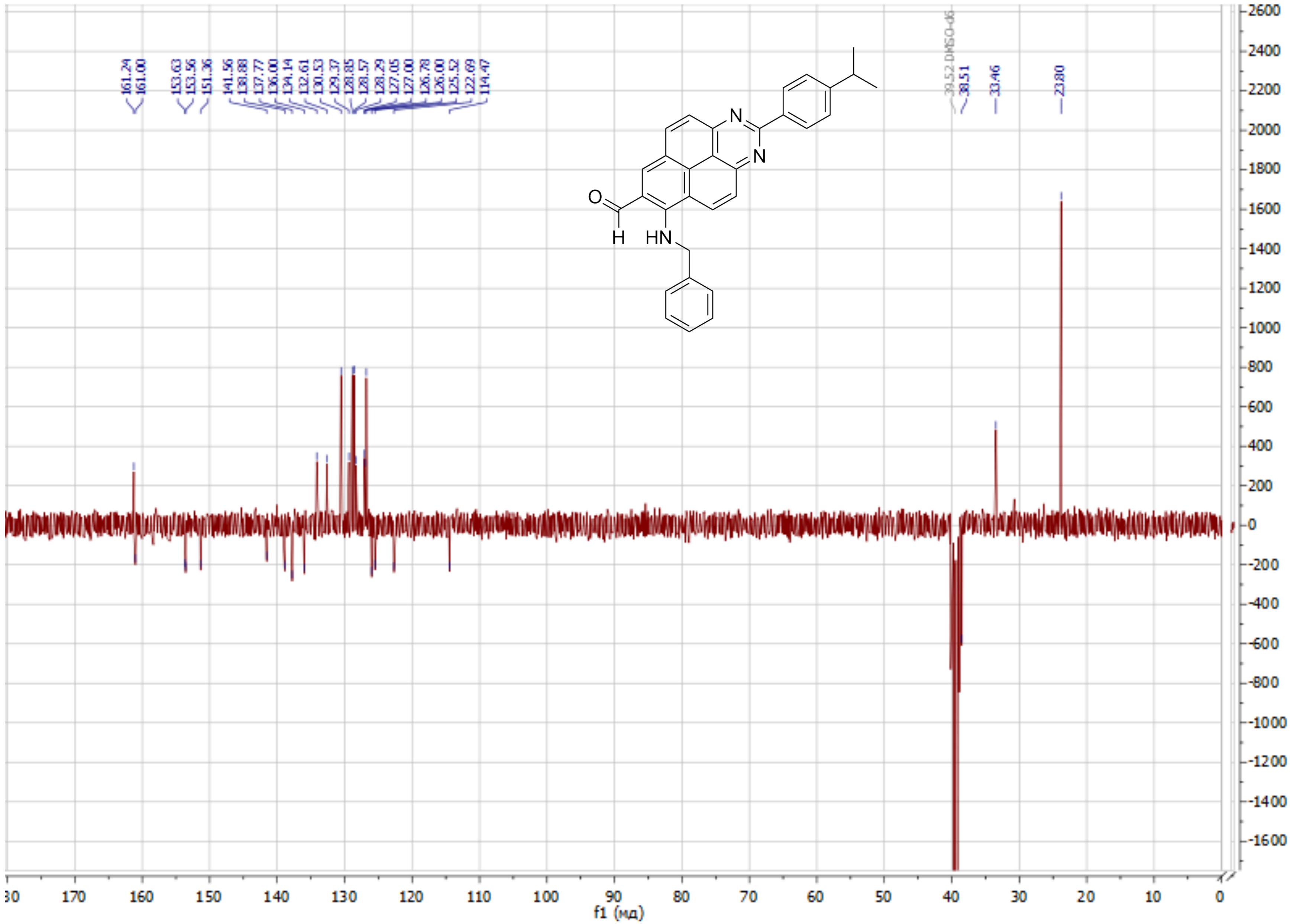

Figure S28. ^{13}C DEPTQ-135 NMR spectrum of **17d** in DMSO-d_6 (101 MHz)

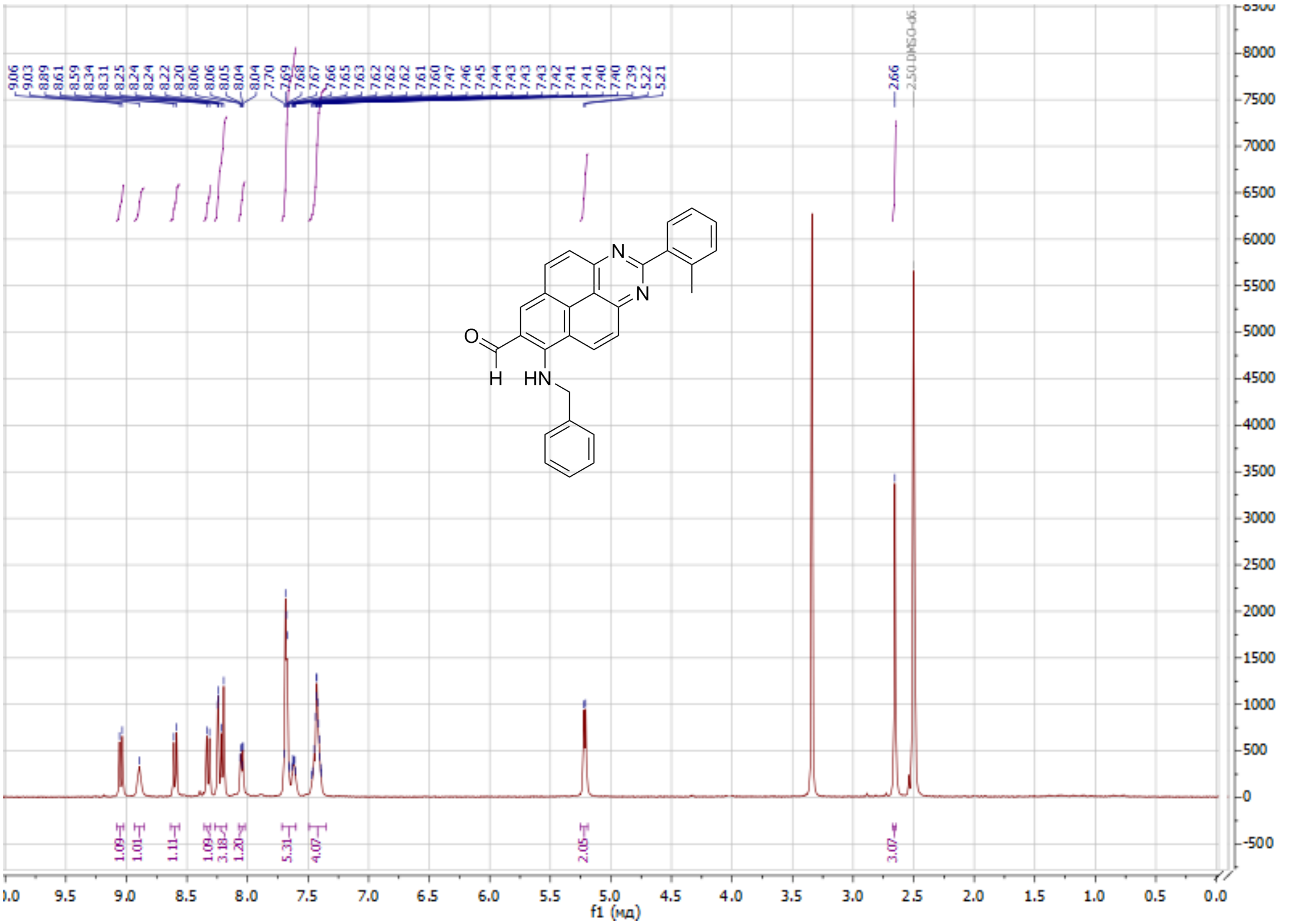

Figure S29. ^{1}H NMR spectrum of **17f** in DMSO-d_6 (400 MHz)

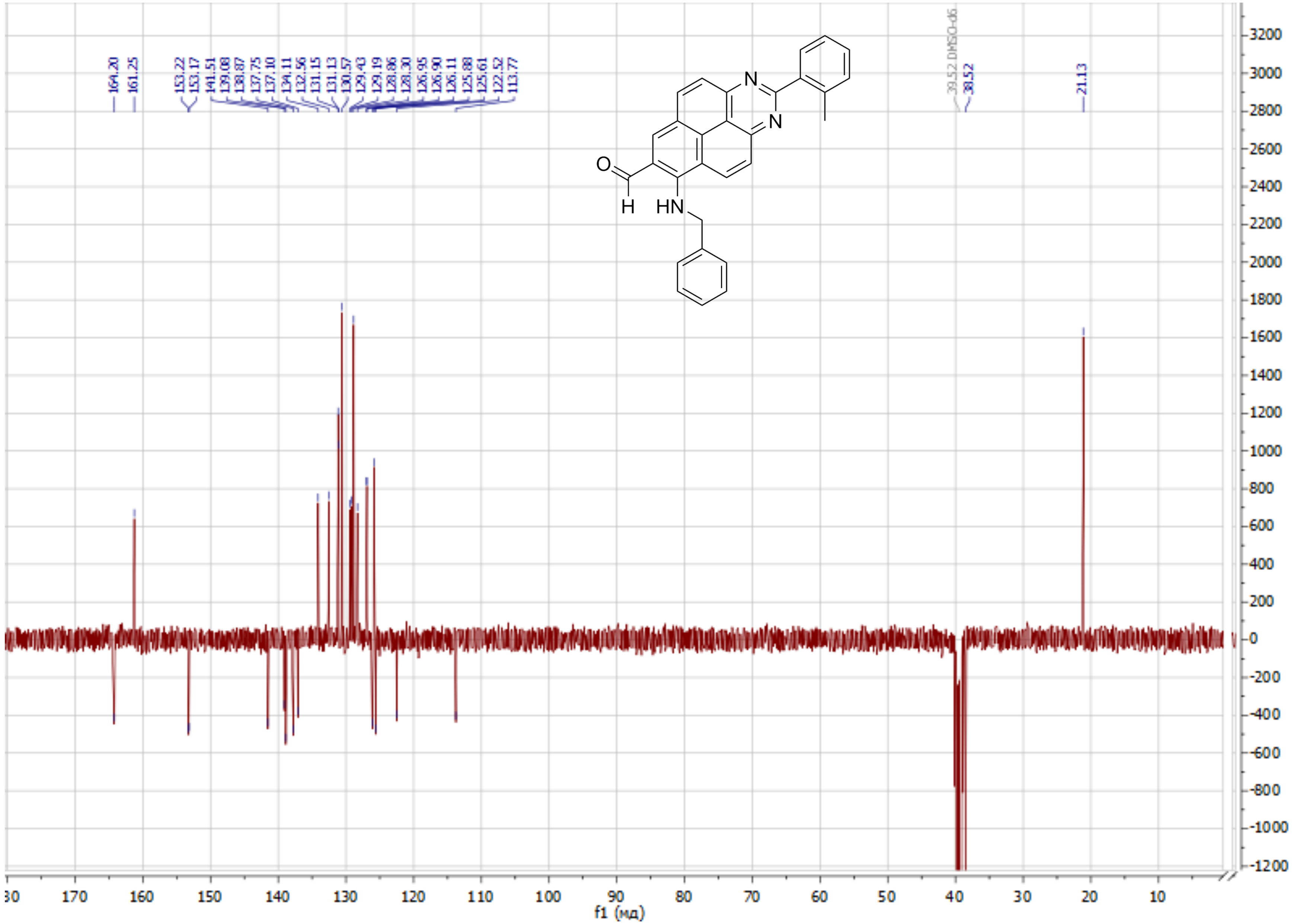

Figure S30. ^{13}C DEPTQ-135 NMR spectrum of **17f** in DMSO-d_6 (101 MHz)

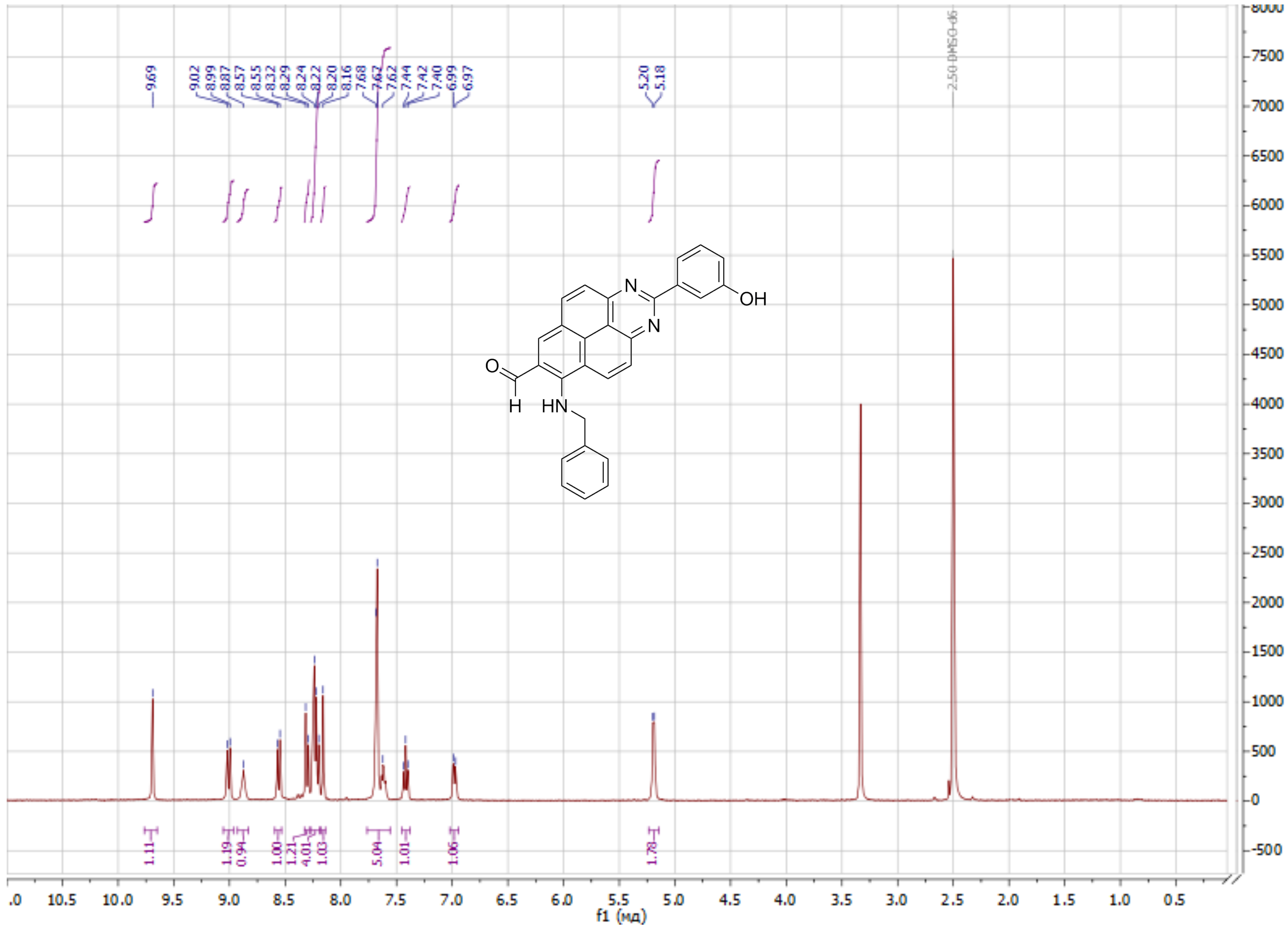

Figure S31. ^{1}H NMR spectrum of **17h** in DMSO-d_6 (101 MHz)

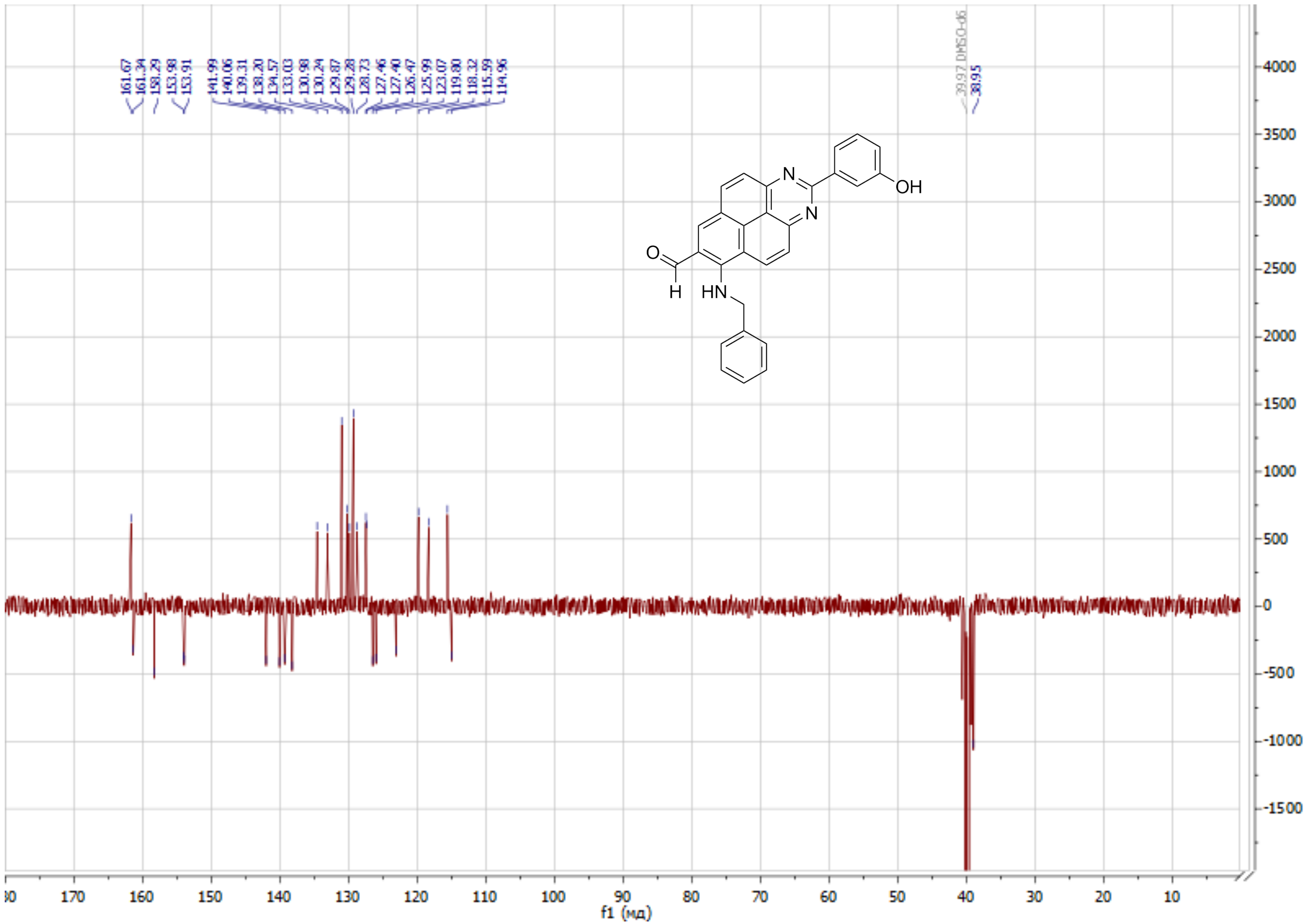

Figure S32. ^{13}C DEPTQ-135 NMR spectrum of **17h** in DMSO-d_6 (101 MHz)

HRMS spectral charts

HRMS spectral charts for 6-benzylidene-6,10,10a,10b-tetrahydroquinazolino[6,7,8-*gh*]perimidine (14)

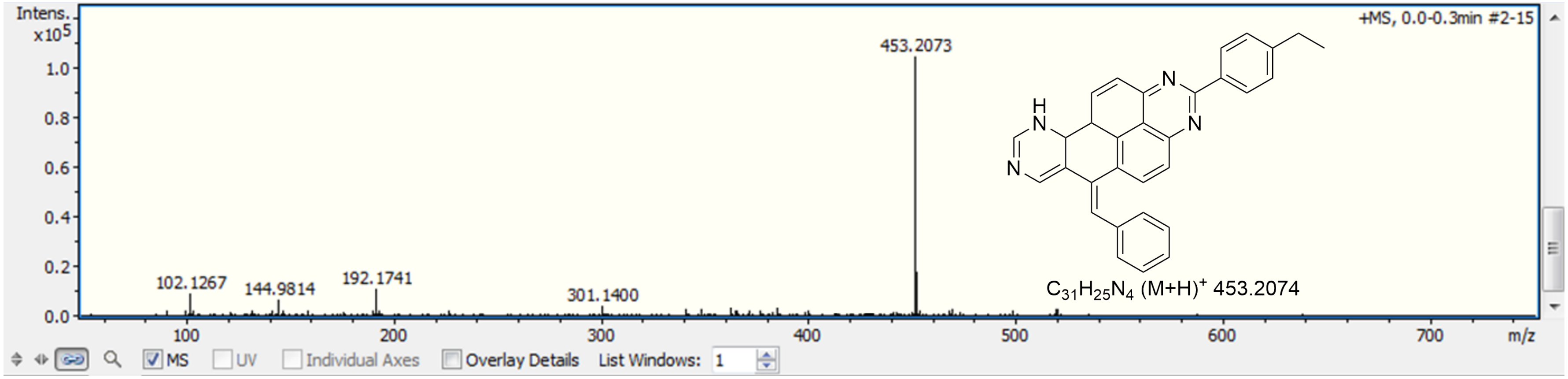

Figure S33. HRMS spectral chart for **14c**

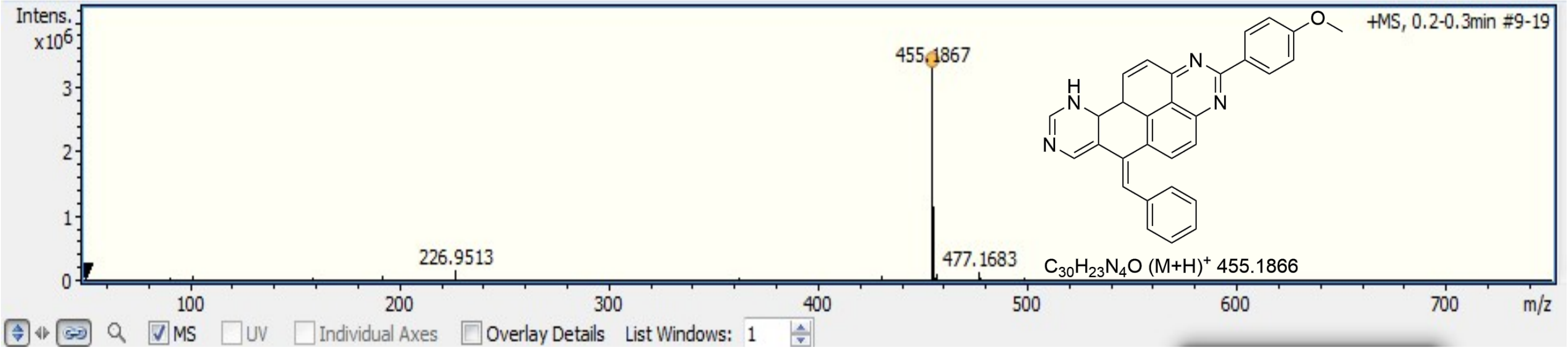

Figure S34. HRMS spectral chart for **14e**

HRMS spectral charts for 6-hexyllbenzo[*gh*]perimidine-7-carbaldehyde (16) and 6-(benzylamino)benzo[*gh*]perimidine-7-carbaldehyde (17)

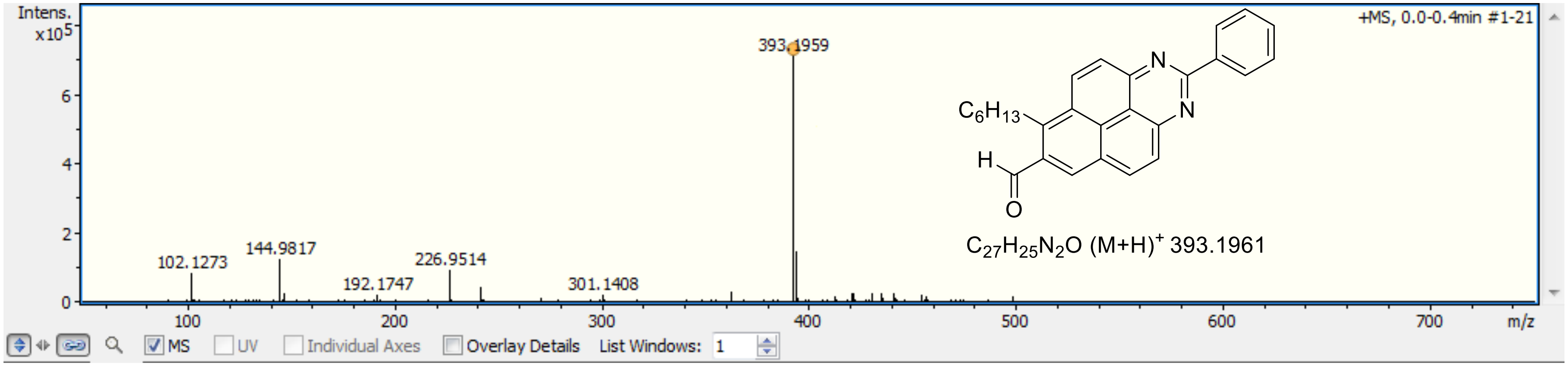

Figure S35. HRMS spectral chart for **16a**

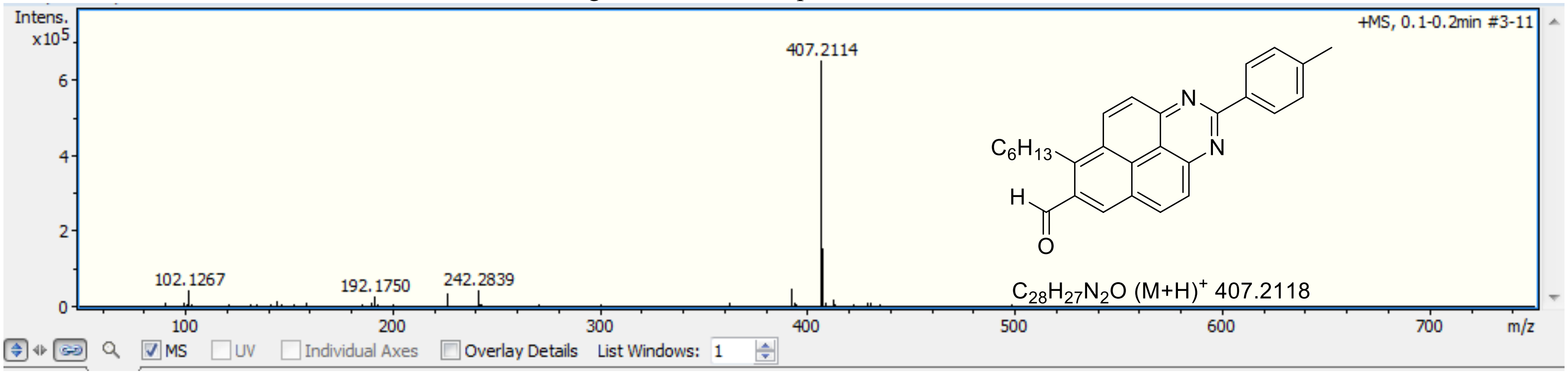

Figure S36. HRMS spectral chart for **16b**

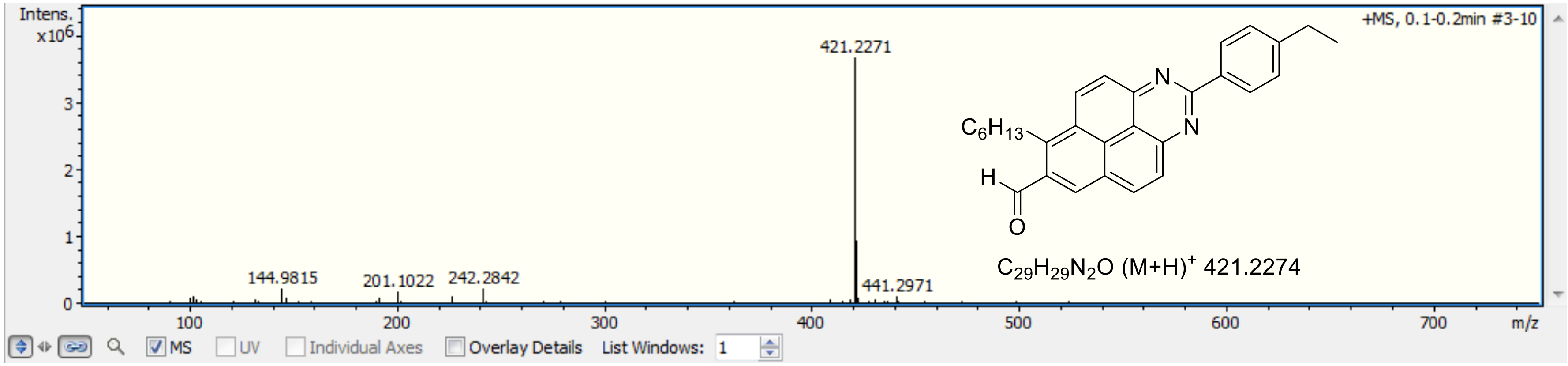

Figure S37. HRMS spectral chart for **16c**

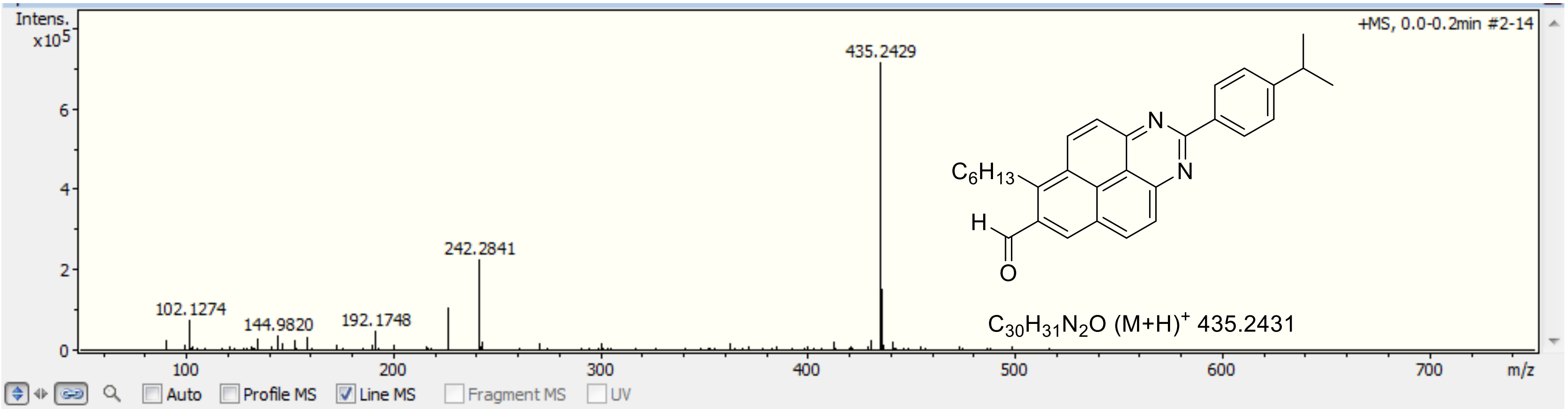

Figure S38. HRMS spectral chart for **16d**

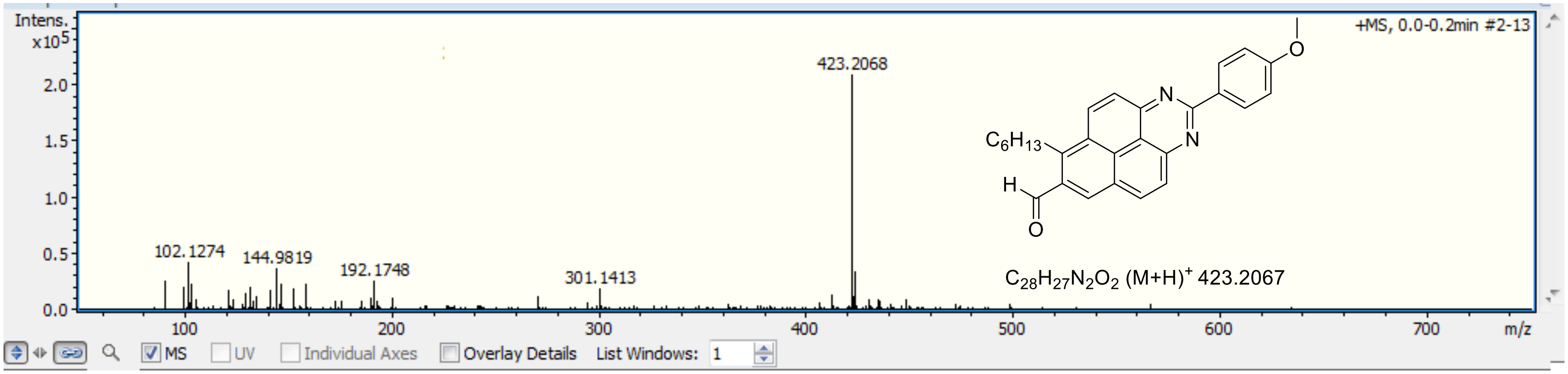

Figure S39. HRMS spectral chart for **16e**

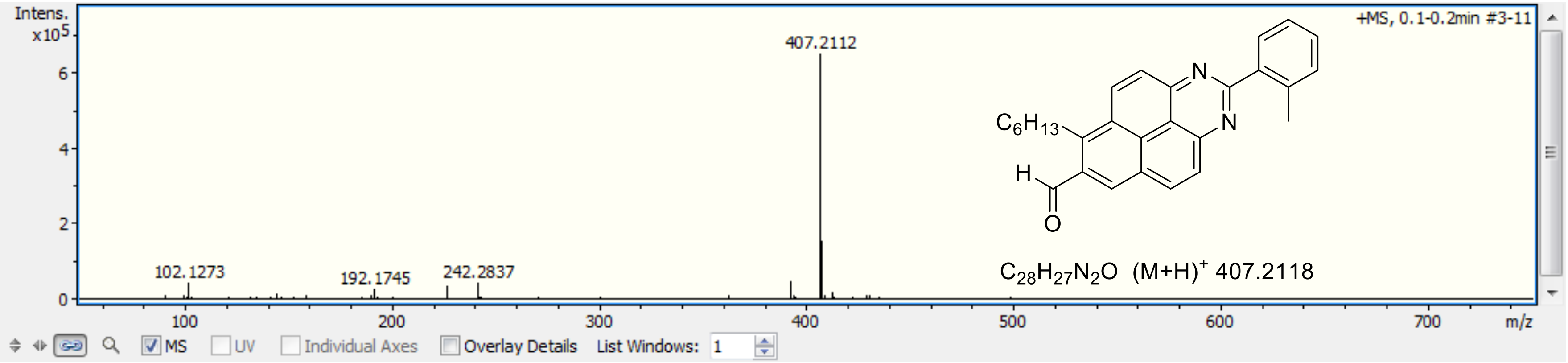

Figure S40. HRMS spectral chart for **16f**

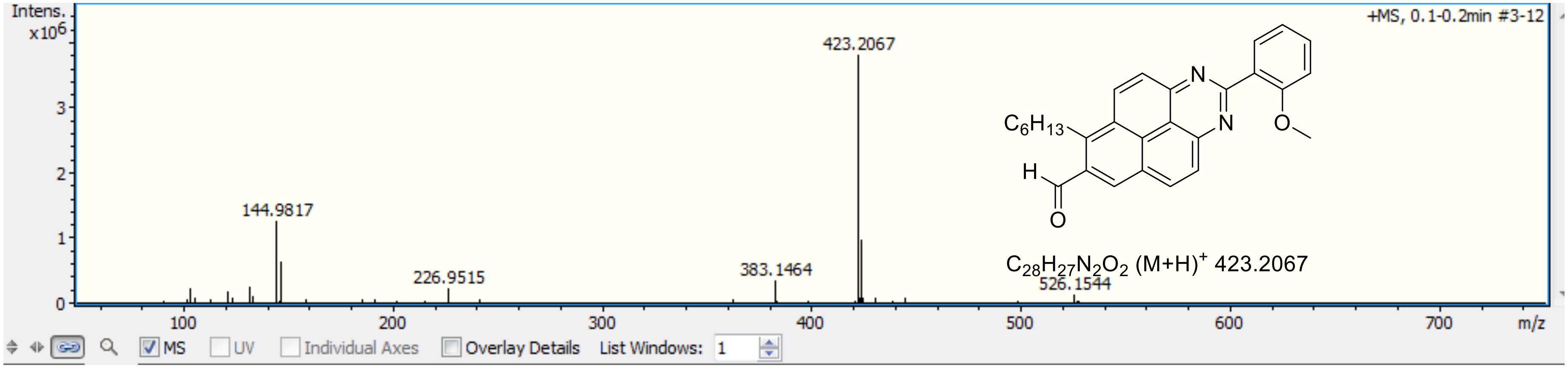

Figure S41. HRMS spectral chart for **16g**

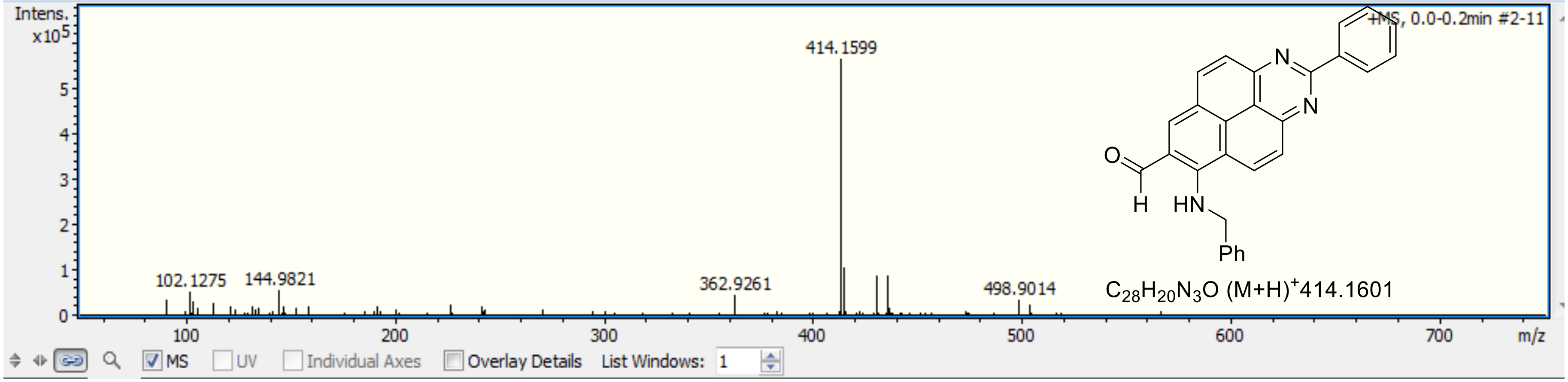

Figure S42. HRMS spectral chart for **17a**

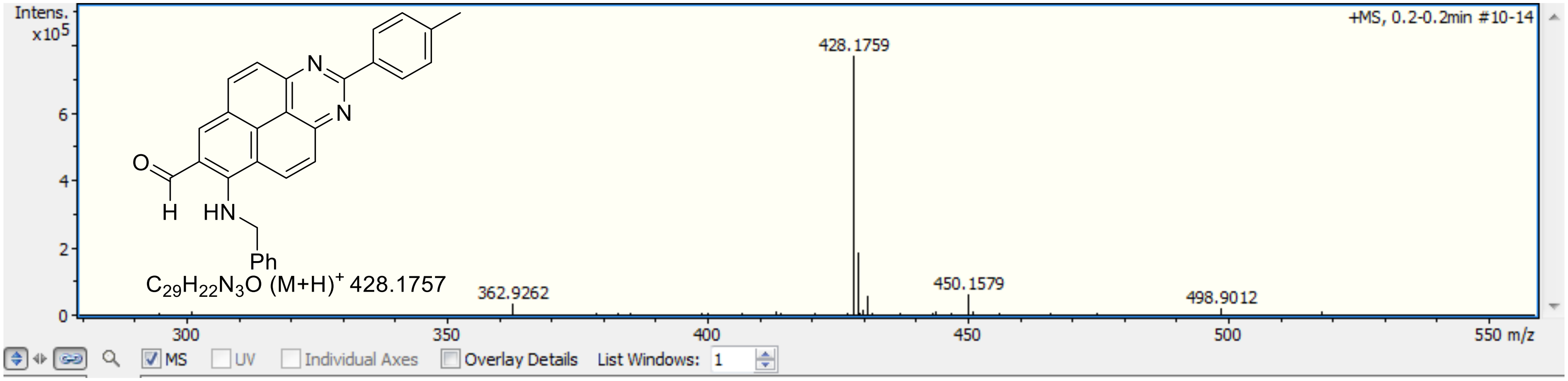

Figure S43. HRMS spectral chart for **17b**

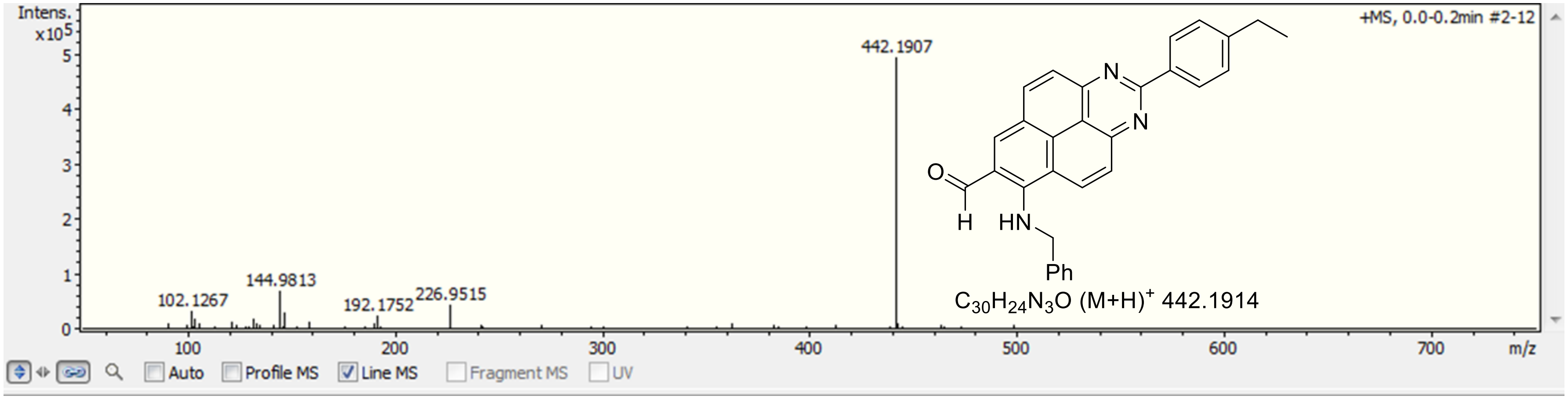

Figure S44. HRMS spectral chart for **17c**

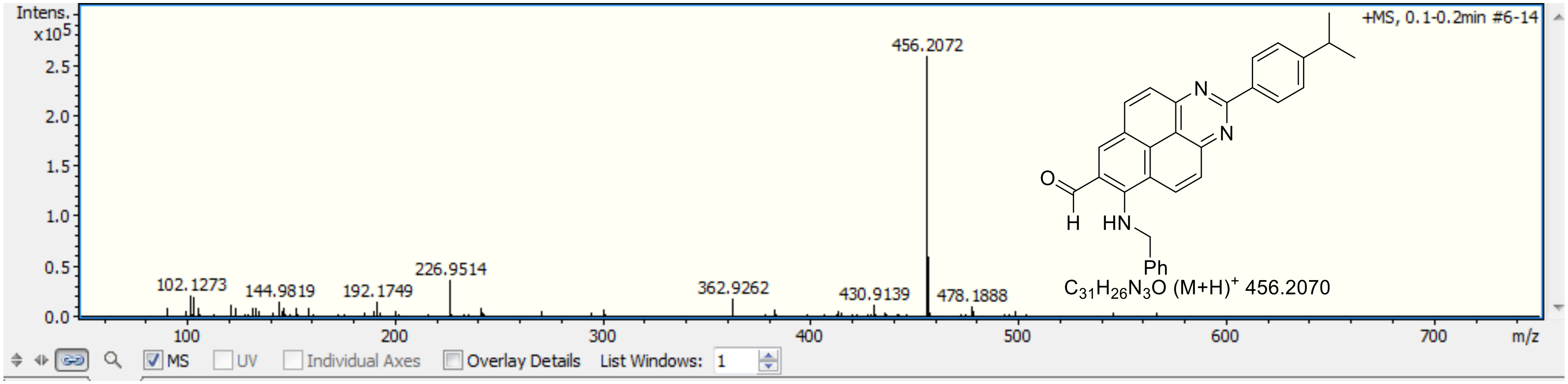

Figure S45. HRMS spectral chart for **17d**

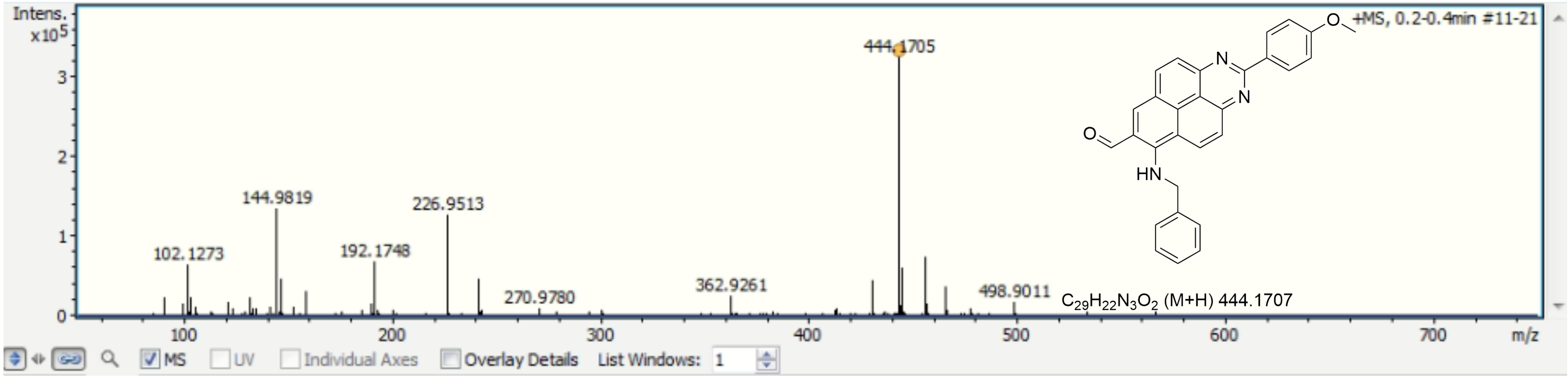

Figure S46. HRMS spectral chart for **17e**

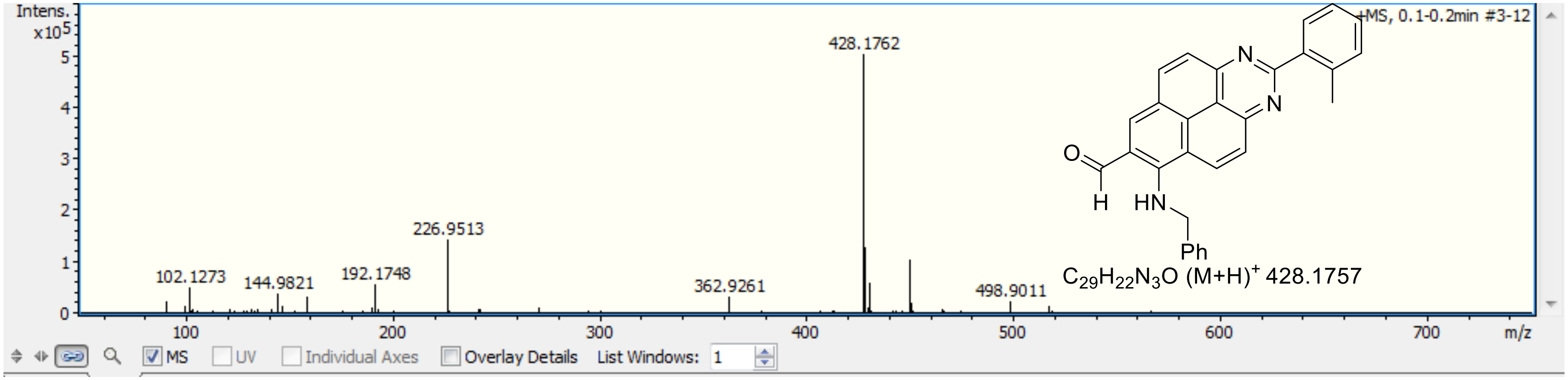

Figure S47. HRMS spectral chart for **17f**

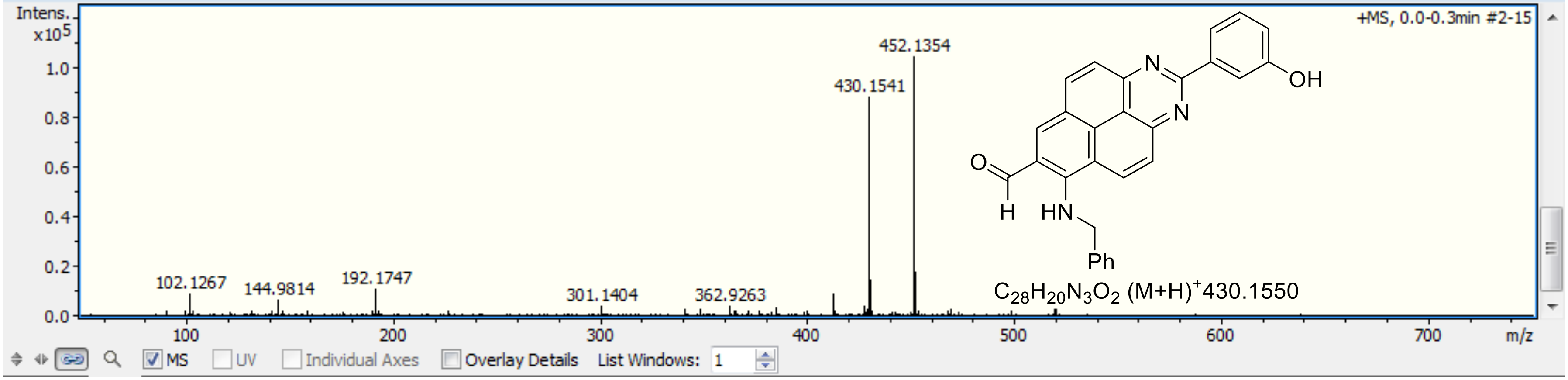

Figure S48. HRMS spectral chart for **17h**

Structure elucidation for compound 17b

Doublet signals in ^{1}H NMR: 9.01 (d, *J* = 9.4 Hz, 1H), 8.56 (d, *J* = 9.4 Hz, 1H), 8.31 (d, *J* = 9.4 Hz, 1H), 8.68 (d, *J* = 7.9 Hz, 2H), 7.43 (d, *J* = 7.9 Hz,2H), are characteristic for AX-systems in 1,3-diazapyrene scaffold as well as 4-tolyl substituent at C-2. Positions C-6 and C-7 should also be substituted (**Figure S49**, **Table S1**).

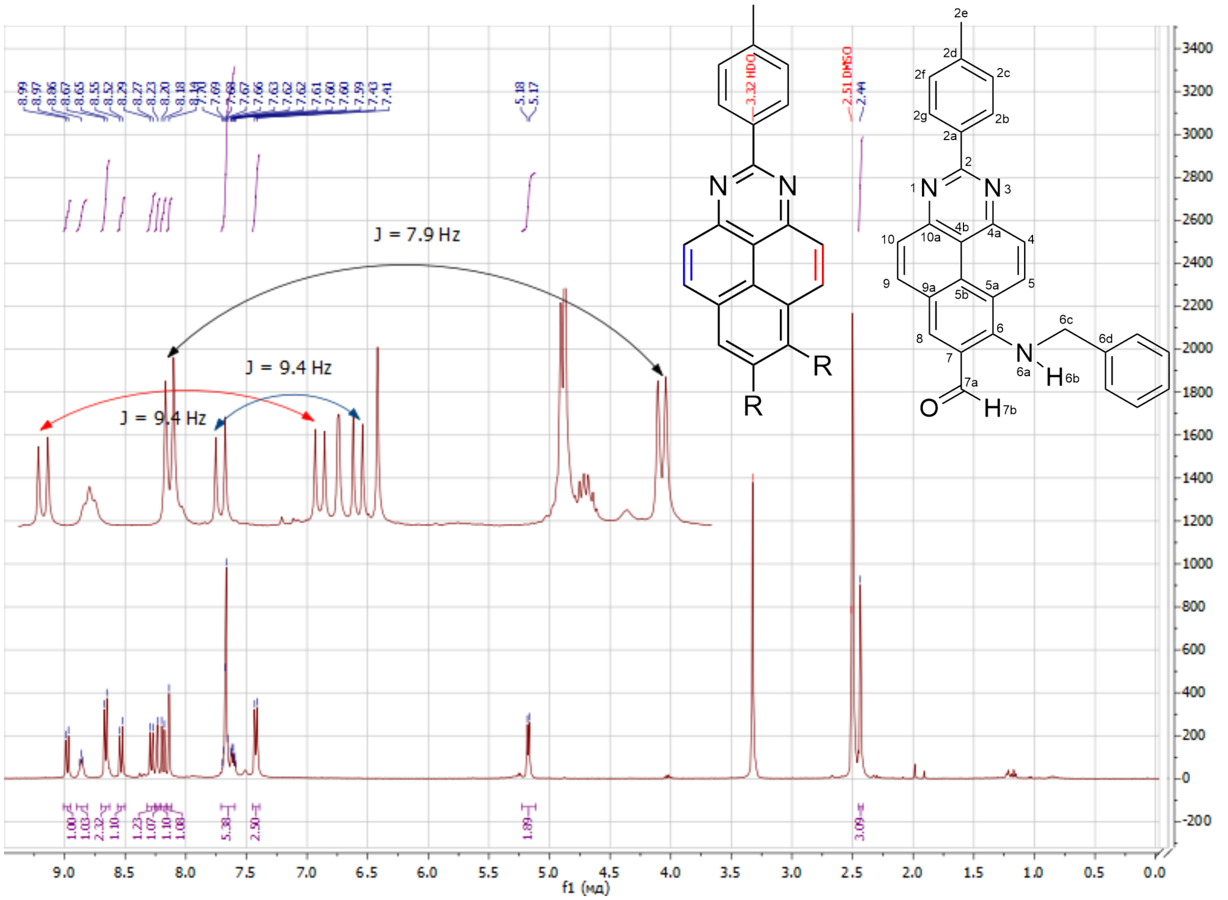

Figure S49 - ^{1}H NMR spectrum of compound **17b**

Table S1. ^{1}H NMR signal assignment for compound 17b.

Chemical shift, ppm	Multiplicity	J, Hz	Integral intensity	Proton number
8.98	d	9.4	1	9
8.86	br.s	-	1	6b
8.66	d	8.1	2	2b, 2g
8.53	d	9.4	1	5
8.28	d	9.4	1	10
8.23	s	-	1	7a
8.19	d	9.4	1	4
8.14	s	-	1	8
7.71 – 7.60	m	-	5	6d-Ph
7.42	d	8.1	2	2c, 2f
5.18	d	6.0	2	6c
2.44	s	-	3	2e

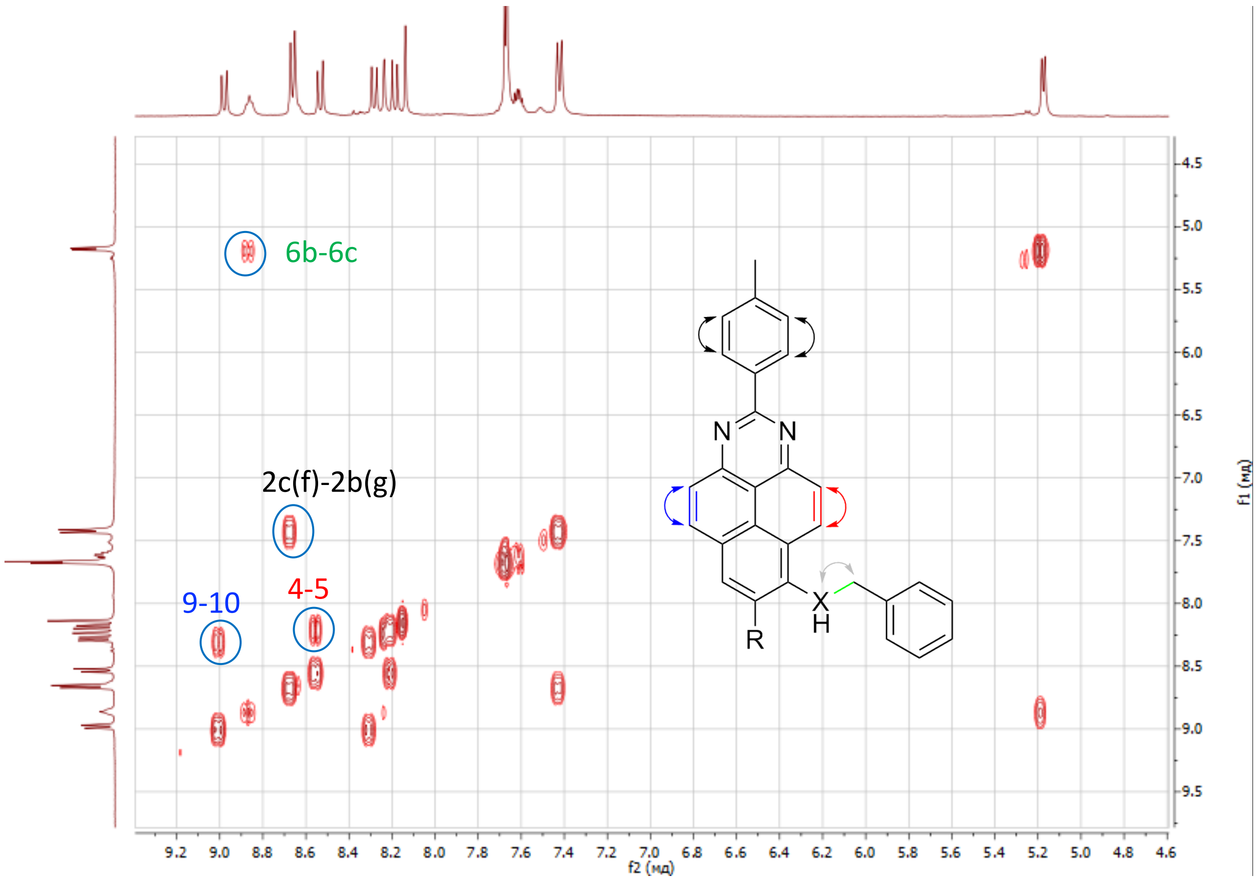

Figure S50. ^{1}H-^{1}H COSY spectrum of compound **17b**

In addition, formation of 1,3-diazapyrene scaffold was confirmed by the presence of characteristic cross-picks in ^{1}H-^{1}H COSY spectrum (Figure S50).

Observed cross-pick at 8.85/5.18 ppm corresponds to correlation between benzylic CH_2 group and broad singlet with integral intensity 1H, which is not connected to any of carbon atoms (as determined from ^{1}H-^{13}C HSQC spectrum, Figure S51, Table S2).

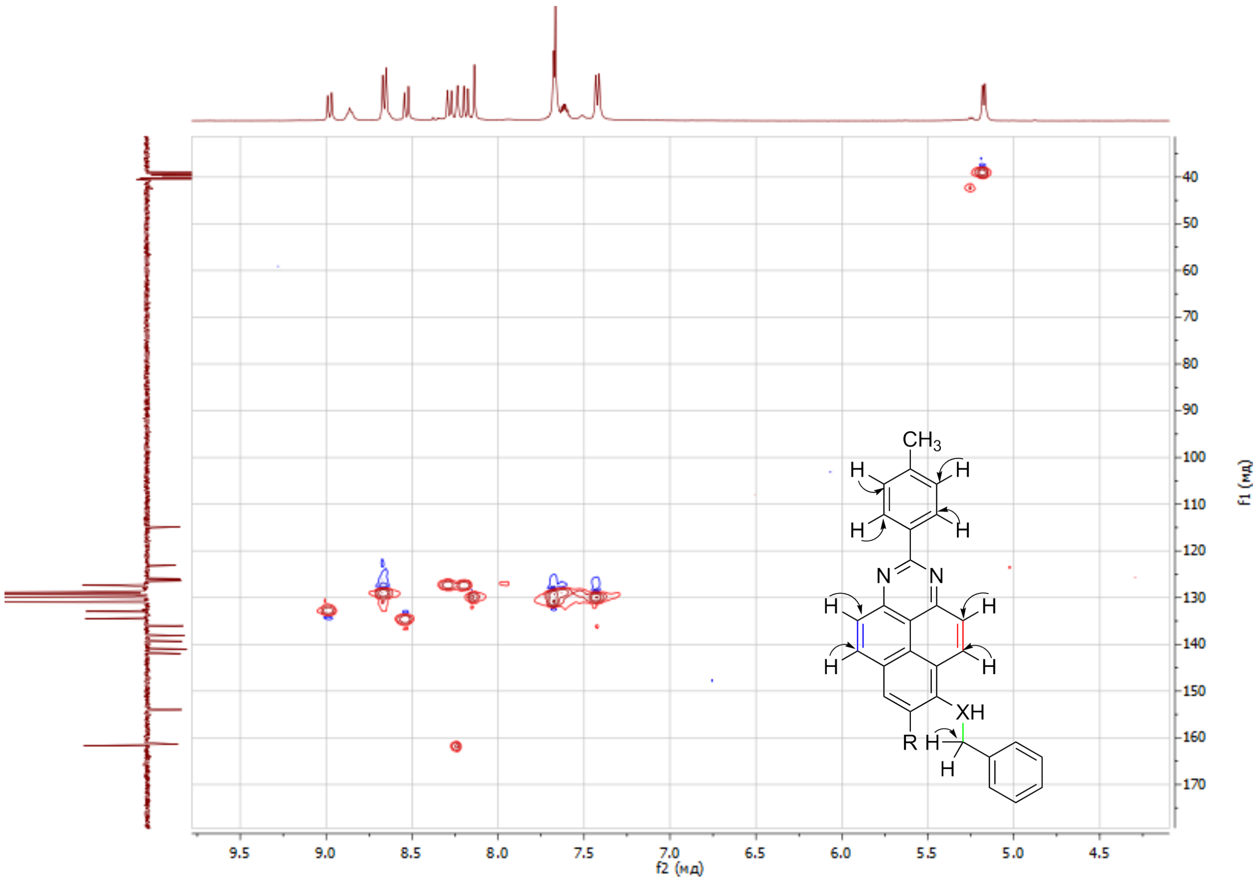

Figure S51 - ^{1}H-^{13}C HSQC NMR spectrum of compound **12e**

Table S2. Correlations ^{1}H-^{13}C HSQC NMR

Proton number	Chemical shift ^{1}H, ppm	Chemical shifts ^{13}C, ppm
9	8.98	134.0
6b	8.86	-
2b, 2g	8.66	128.8
5	8.53	132.5
10	8.28	127.3
7a	8.23	161.2
4	8.19	126.9
8	8.14	129.3
6d-Ph	7.71 – 7.60	-
2c, 2f	7.42	128.4
6c	5.18	38.5
2e	2.44	21.1

Spectrum ^{1}H-^{15}N HSQC NMR of compound **17b** shown in Figure S52 suggests that this proton is linked to a nitrogen atom with chemical shift at 122.3 ppm.

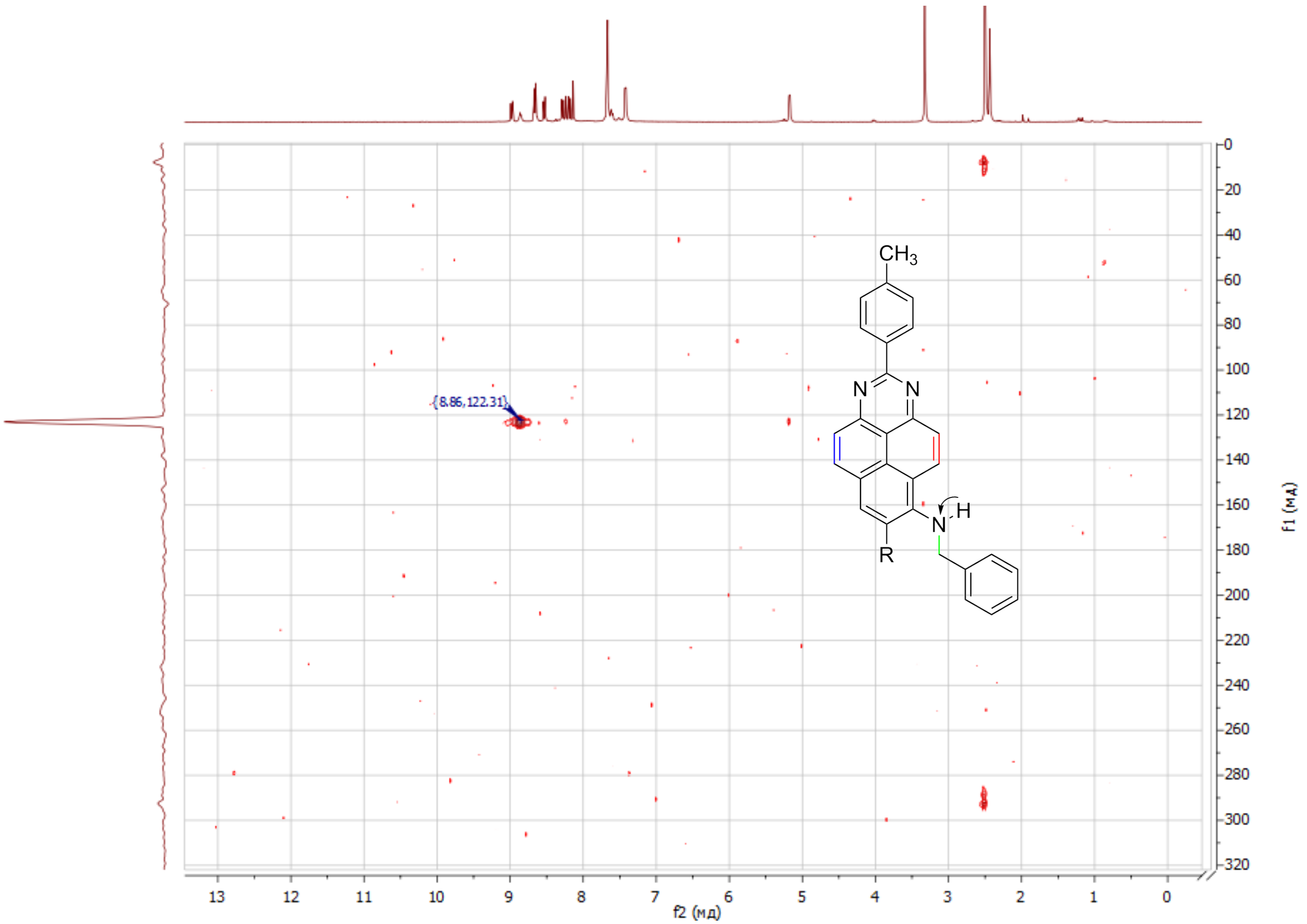

Figure S52 - ^{1}H-^{15}N HSQC NMR spectrum of compound **17b**

Correlations between amine nitrogen atom and doublet at 5.18 ppm (benzylic group) and singlet at 8.25 ppm (aldehyde function) was established by ^{1}H-^{15}N HMBCGP NMR spectrum (Figure S53). Presence of the aldehyde functionality was also confirmed by FT-IR spectrum (Figure S53).

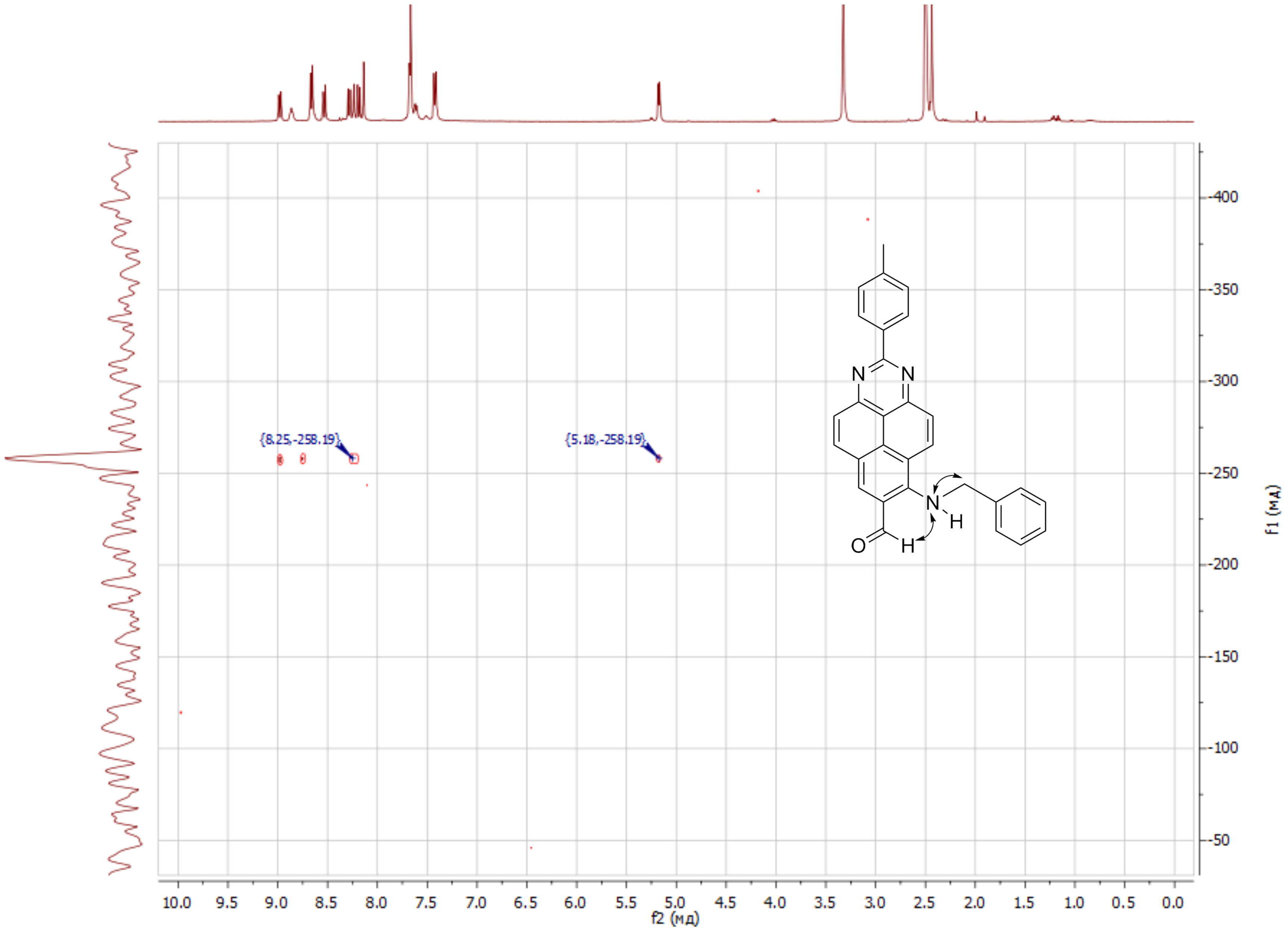

Figure S53. ^{1}H-^{15}N HMBCGP NMR spectrum of compound **17b**

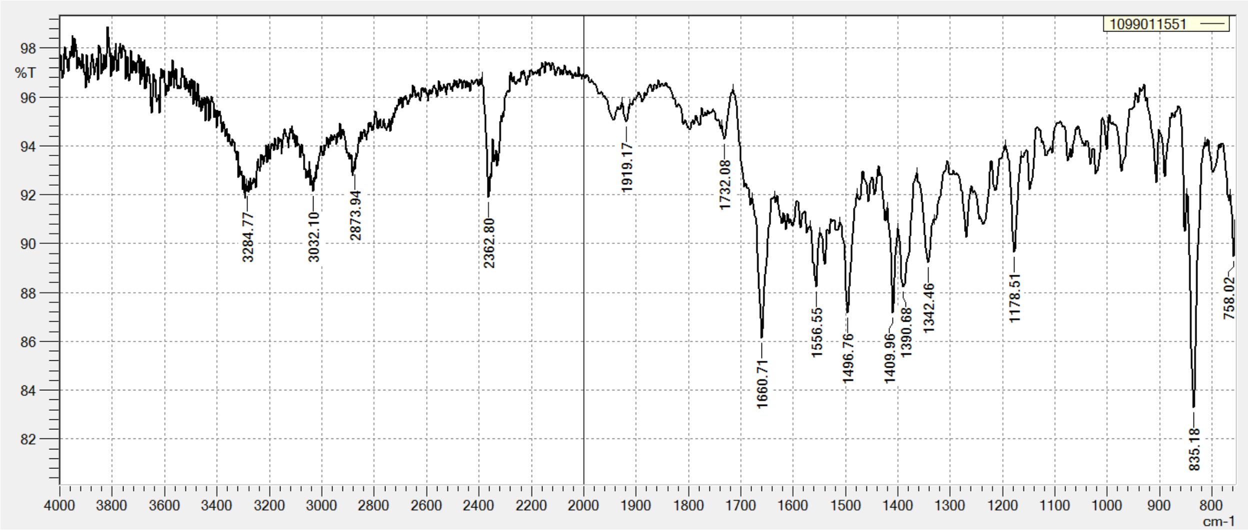

Figure S54. FT-IR spectrum of compound **17b**

Also, structure of compound **17b** was elucidated by correlations in ^{1}H - ^{13}C HMBC NMR spectrum (Table S3, Figure S55).

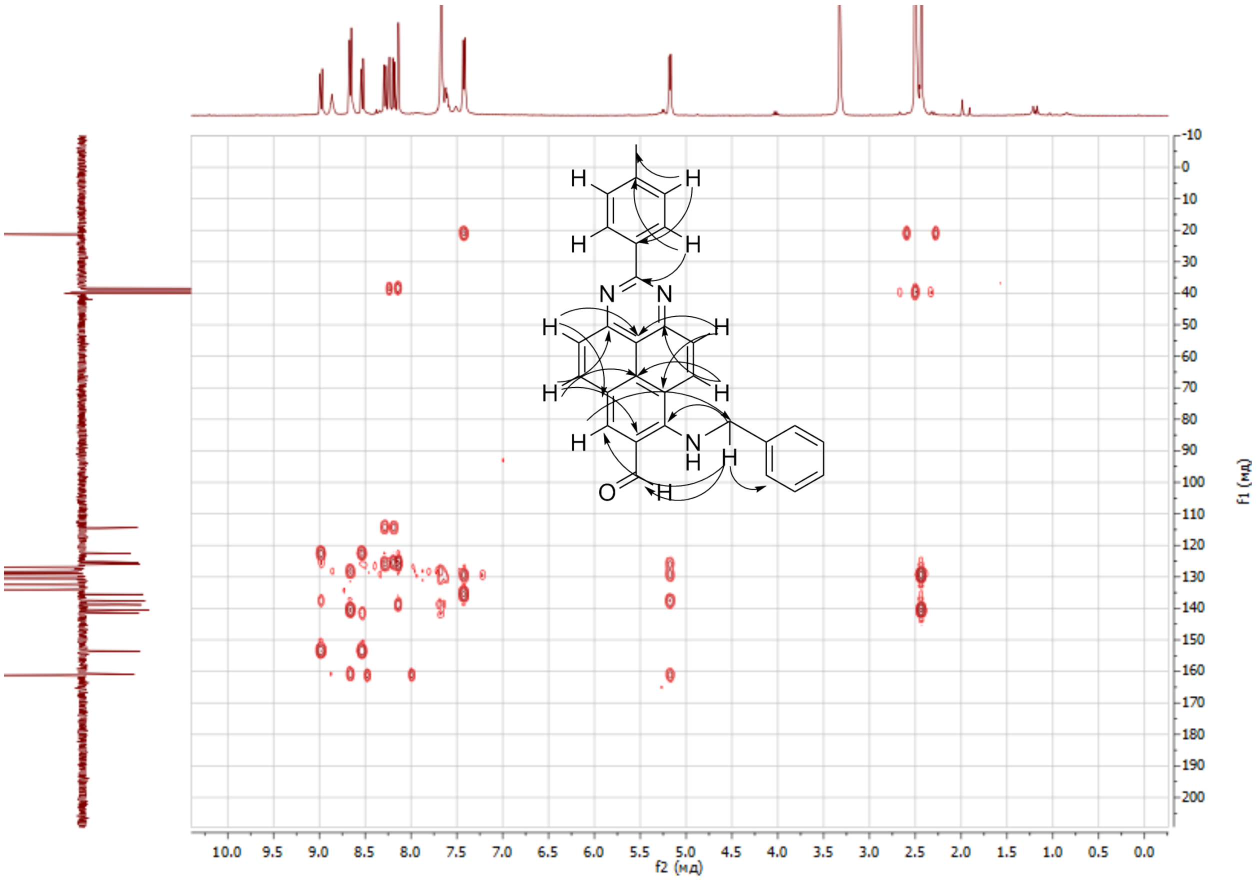

Figure S55. ^{1}H-^{13}C HMBC NMR spectrum of compound **17b**

Table S3. Signal assignment in ^{1}H -^{13}C HMBC spectrum of compound **17b**.

**Atom number**	**Chemical shifts ^{1}H, ppm**	**Chemical shifts ^{13}C, ppm**				
		+	+	-	-	-
**9**	8.98			153.5	137.7	122.6
**6b**	8.86					
**2b, 2g**	8.66				160.9	140.5
**5**	8.53			153.5	141.5	122.6
**10**	8.28				114.4	125.9
**7a**	8.23				38.5	
**4**	8.19				114.4	125.5
**8**	8.14			38.5	125.5	138.9
**6d-Ph**	7.71 – 7.60	-	-	-	-	-
**2c, 2f**	7.42	21.1	128.8		135.6	
**6c**	5.18	161.2	128.9		137.7	126.0
**2e**	2.44	128.4			140.5	
